# Allosteric Communication Occurs via Networks of Tertiary and Quaternary Motions in Proteins

**DOI:** 10.1371/journal.pcbi.1000293

**Published:** 2009-02-20

**Authors:** Michael D. Daily, Jeffrey J. Gray

**Affiliations:** 1 Program in Molecular & Computational Biophysics, Johns Hopkins University, Baltimore, Maryland, United States of America; 2 Department of Chemical & Biomolecular Engineering, Johns Hopkins University, Baltimore, Maryland, United States of America; University of California San Francisco, United States of America

## Abstract

Allosteric proteins bind an effector molecule at one site resulting in a functional change at a second site. We hypothesize that allosteric communication in proteins relies upon networks of quaternary (collective, rigid-body) and tertiary (residue–residue contact) motions. We argue that cyclic topology of these networks is necessary for allosteric communication. An automated algorithm identifies rigid bodies from the displacement between the inactive and the active structures and constructs “quaternary networks” from these rigid bodies and the substrate and effector ligands. We then integrate quaternary networks with a coarse-grained representation of contact rearrangements to form “global communication networks” (GCNs). The GCN reveals allosteric communication among all substrate and effector sites in 15 of 18 multidomain and multimeric proteins, while tertiary and quaternary networks exhibit such communication in only 4 and 3 of these proteins, respectively. Furthermore, in 7 of the 15 proteins connected by the GCN, 50% or more of the substrate-effector paths via the GCN are “interdependent” paths that do not exist via either the tertiary or the quaternary network. Substrate-effector “pathways” typically are not linear but rather consist of polycyclic networks of rigid bodies and clusters of rearranging residue contacts. These results argue for broad applicability of allosteric communication based on structural changes and demonstrate the utility of the GCN. Global communication networks may inform a variety of experiments on allosteric proteins as well as the design of allostery into non-allosteric proteins.

## Author Summary

Allosteric regulation is a major mechanism of control in many biological processes, including cell signaling, gene regulation, and metabolic regulation, and malfunctioning allosteric proteins are often involved in cancer and other diseases. In allostery, an effector-binding signal transmits over a long distance through the protein structure, resulting in a functional change at a second site. While many three-dimensional structures of allosteric proteins have been solved, the allosteric communication mechanism is usually not obvious from the motions between inactive and active state structures. In addition, allosteric structural transitions involve both small-scale motions at the level of amino acid residues and large-scale motions at the level of domains. Here, to address allosteric mechanisms, we transform the aforementioned protein motions into a multi-scale “global communication network” (GCN) representation from which substrate-effector pathways and other important allosteric communication properties can be identified. The GCN accounts for substrate-effector pathways in 15 of 18 proteins surveyed, and the GCN reveals that allostery often depends on linkage between the small- and the large-scale motions. This work will inform a wide variety of experiments investigating allostery, and it proposes concepts for engineering allostery into non-allosteric proteins.

## Introduction

The modern concept of allostery began with the models of Monod et al. (MWC model) [1] and Koshland et al. (KNF model) [2], which sought to account for allostery based upon gross properties of the transition between two well-defined end-states. More recent thermodynamic models of allostery characterize population shifts in conformational ensembles in more detail [3]–[5], and there is experimental evidence that alternate allosteric states are simultaneously populated in solution [6],[7]. Nonetheless, mechanical and chemical transitions in individual molecules underlie the thermodynamic properties of allosteric proteins. That is, in individual molecules, energetic pathways of spatially contiguous, physically coupled structural changes and/or dynamic fluctuations must link substrate and effector sites [8]–[10].

Crystal structures have revealed that most allosteric proteins are complex systems with both tertiary and quaternary structural changes [11]. Previously, we quantified allosteric communication through tertiary structure from graphs of residue-residue contacts that form, break, or rearrange in the transition between inactive and active state structures [12]. In such network representations of protein structure, putative paths between residues distant in three-dimensional space can be readily identified. These tertiary networks or “contact rearrangement networks” (CRNs) identified substrate-effector paths in 6 of 15 proteins tested, which indicated that tertiary changes play a significant but incomplete role in allosteric communication. In this work, we broaden the CRN approach toward more completely quantifying allosteric coupling mechanisms from structure. Specifically, we develop a network representation of quaternary structural changes (collective / rigid-body motions) and integrate this representation with the CRN.

We seek to infer information about the allosteric coupling mechanism from gross properties of the differences between inactive and active structures. In this, our work resembles the MWC [1] and KNF [2] approaches but differs from investigations of the kinetic mechanism, that is, the order of events in the transition between inactive and active structural regimes [13]–[16]. Most current computational approaches to large-scale protein dynamics (e.g. normal mode analyses [17]–[20], Gō models [21], and all-atom simulations [22],[23]) predict motions and/or associated energetics by applying to the structure(s) theoretical models like the elastic network [24] and potential functions. While these predictions address important problems, most of these approaches do not predict allosteric pathways. By contrast to these problems, we will argue that allosteric pathway identification is facilitated by a network representation of a protein structural transition.

Network representations of protein structures have previously been used to illuminate dynamic and/or allosteric properties. For example, large-scale fluctuations predicted from normal mode analysis of the elastic network correlate with known conformational changes [17],[19],[25]. In addition, rigid and flexible regions of protein structures have been predicted from the network of contact and hydrogen bond constraints in a single protein structure [26],[27]. Furthermore, residues important for maintaining short paths in a contact network are experimentally known to mediate signaling in proteins [28]. However, allosteric communication pathways have not previously been derived from a network representation of the quaternary structural transition.

In this paper, we develop a hypothesis for allosteric coupling via networks of quaternary motions. We elucidate rigid bodies from the differences between inactive and active crystal structures with an automatic algorithm, and we form a “quaternary network” from the rigid bodies based on contacts between them. Toward a broader representation of allosteric communication mechanisms, we assess how communication through these networks relates to that through contact rearrangement networks in tertiary structure. We then integrate quaternary networks with a coarse-grained representation of CRNs to form “global communication networks” (GCNs). We describe the range of topologies of GCNs in several representative proteins from the allosteric benchmark set [29], and then we assess substrate-effector communication via CRNs, the quaternary network, and the GCN in 18 DNA-binding proteins and enzymes (including the 15 assessed by the CRN [12]) and classify each protein based on the respective tertiary and quaternary contributions to connectivity. GCN analysis provides the opportunity to advance the theory of mechanical allosteric coupling in proteins and may guide drug design and allosteric experiments and simulations.

## Results

### Theory

**Figure 1:**
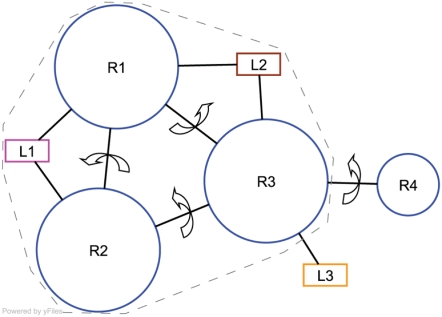
Allosteric coupling via quaternary motions. In this sample graph, circular nodes represent “rigid body” groups of residues that move collectively, and rectangular nodes represent ligands. Circular node area is proportional to physical size in number of residues. Edges represent motions between physically contacting rigid bodies. A grey dashed line marks the boundary of the allosteric unit.

Figure 1 shows a sample quaternary network (QN) representing the rigid-body motions in the allosteric transition. Rigid bodies can range in size from single secondary structure elements to domains to entire subunit or multisubunit cores. Our analysis is entirely geometric and we do not estimate any energetics to create or interpret our model. Presumably, the relative displacement between two contacting rigid bodies would be accompanied by (and/or driven by) underlying changes in the energetics of residues interacting across the interface.

We hypothesize (“the cyclic connectivity hypothesis,” CCH) that mechanical coupling via quaternary motions can occur only within cyclic substructures of a QN such as the R1-R2-R3 cycle of Figure 1. In such a cycle, any motion (e.g. R1-R2) necessitates at least one other co-cyclic motion (e.g. R2-R3 and/or R1-R3) because the internal (i.e. relative rotational and translational) coordinates of co-cyclic motions are coupled to one another. By extension, in a cycle of motions, an initial perturbation will trigger a series of compensating perturbations until the system equilibrates to a new conformational state. By contrast, the internal coordinates of an exocyclic motion (e.g. R3-R4) are independent of the configuration of the rest of the QN; that is, an exocyclic motion can be achieved without moving any of the other degrees of freedom in the system. In graph theoretic terms, the CCH entails that the allosterically connected subsets or “allosteric units” of a QN must be at least 2-connected, where a graph is *k*-connected if at least *k* nodes must be removed to disconnect it. In addition to an exocyclic portion of a QN, a 1-connected cyclic graph is allosterically disconnected (see Figure S1). In addition, we hypothesize that a ligand (e.g. L1 or L2) can participate in an allosteric unit by binding to at least two rigid bodies in the QN because a motion between two such rigid bodies would perturb the ligand-binding site. That is, a ligand can be part of an allosteric unit. By this hypothesis, the allosteric unit of the Figure 1 QN is the R1-R2-R3 cycle plus L1 and L2, while R4 and L3 are allosterically isolated. Therefore, ligands L1 and L2 are allosterically coupled.

### Rigid-Body Identification and Quaternary Network

To identify allosterically coupled units of quaternary motions in real proteins, we first create maps of the quaternary motions inferred by comparing inactive and active structures. An automated approach similar to previous studies [30]–[32] is used to identify rigid bodies from a comparison of the coordinates of two structures. However, before identifying rigid bodies, “flexible segments,” that is, segments with significant local backbone conformational change, are removed. Our algorithm is detailed in the methods.

**Figure 2:**
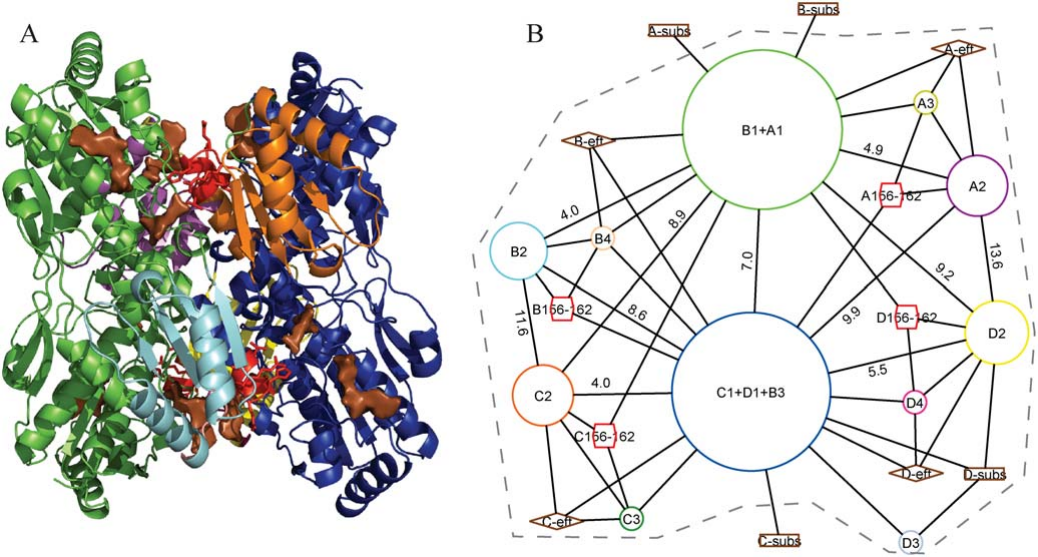
Rigid-body partitioning and quaternary network in phosphofructokinase (PFK). (A) The active state structure (4PFK) colored by identified rigid bodies, except red, which marks flexible segments. (B) Quaternary network representation of the quaternary nodes (rigid bodies and flexible segments) shown in (A). Circular nodes represent rigid bodies and hexagonal nodes represent flexible segments. Areas of protein nodes correspond to their physical sizes in number of residues, and their colors correspond to the colors of the rigid bodies in (A). Each rigid body is labeled by the chains and domains it contains, e.g. the rigid body labeled “A1+B1” contains the largest portion of chain A and the largest portion of chain B, the rigid body labeled “D2” contains the second-largest portion of chain D, etc. Each flexible segment is labeled by its chain identifier followed by its range of residue numbers. Substrate and effector “sites” are shown as rectangles and diamonds, respectively. Each substrate (effector) site represents all the substrate (effector) molecules from a given chain in either the inactive or the active state. An edge indicates a quaternary interface, that is, two or more atomic (4.0 Å) contacts between a pair of quaternary nodes, or two or more atomic contacts between a quaternary node and a ligand. An edge between two rigid bodies is labeled by the rotation in degrees (see methods for rotation calculations), provided the smaller rigid body is ten residues or larger. A grey dashed line marks the boundary of the main allosteric unit of the graph. Graphs drawn by yEd graph editor (http://www.yworks.com).

Figure 2A shows the rigid bodies and flexible segments identified for the tetrameric protein phosphofructokinase (PFK). The largest rigid bodies are the two dimer cores (chains A+B and chains C+D), shown in green and blue, respectively. In addition, in each subunit, a small domain moves relative to the dimer core (small domains in purple, yellow, orange, and cyan), and a flexible segment (red) undergoes a large distortion in local conformation. The algorithm also groups a small portion of chain B with the dimer core of chains C and D, a boundary error that probably results from the uncertainties of the crystal structure. The interdimer motion is supported by the previous manual analysis by Schirmer and Evans from their crystal structures [33]. Additionally, our approach identified the collectivity of the motion of the small domains.

Figure 2B shows the quaternary network (QN) representation of the rigid bodies and flexible segments (both are “quaternary nodes”) shown in Figure 2A. The quaternary nodes C1+D1+B3 and A1+B1 correspond to the two main dimer cores, while nodes A2, B2, C2, and D2 correspond to the smaller domains of each subunit. Major rigid-body motions include a rotation of 7.0° between the two dimer cores, a rotation between the small and large domains of each subunit ranging from 4–6° depending on the subunit, a rotation of 8.5–10° between the small domain of each subunit and the opposing dimer core, and a rotation of 12–14° between the small domains A2 and D2 and also for the symmetrically matching pair B2-C2. Some of these rigid-body motions may also involve significant translation, but since the meaning of rigid-body translation depends on the position of the center of rotation, we do not show translations in Figure 2. Rather, we treat rigid-body translations and their relationship to rotation later. Finally, the cyclic connectivity hypothesis (see theory section) identifies a single allosteric unit of the PFK QN (encircled with grey dashed lines). This allosteric unit includes all four effector sites but only the substrate of chain D, while the remaining three substrate sites are exocyclic. This incomplete allosteric connectivity via the quaternary network of PFK suggests the possible importance of other kinds of structural changes, such as tertiary changes, to allosteric connectivity.

### How Do Tertiary and Quaternary Communication Relate?

**Figure 3:**
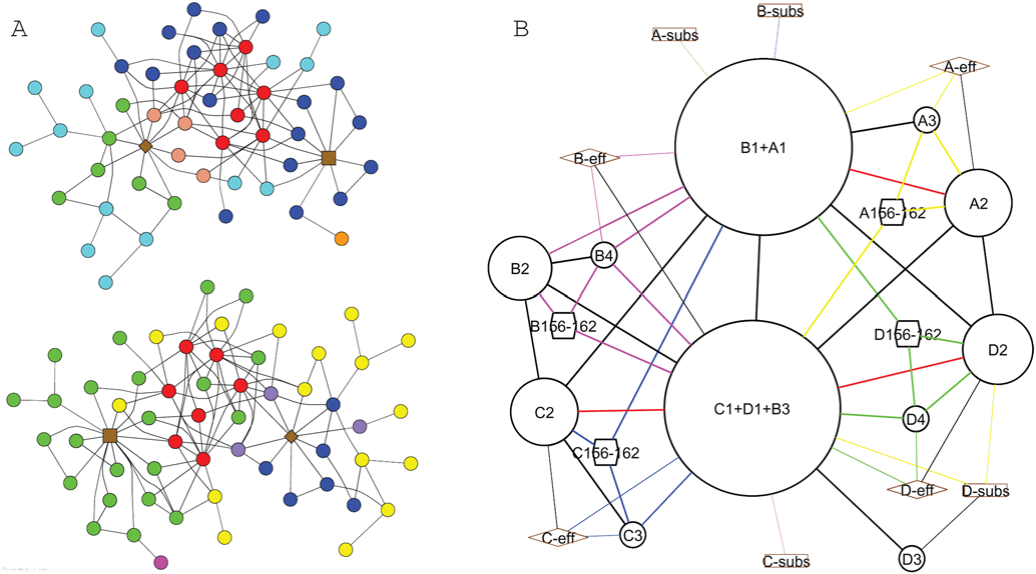
Relationship of tertiary and quaternary communication in PFK. (A) Contact rearrangement network (CRN) of phosphofructokinase. As described previously [12], nodes are protein residues (circles), effector sites (diamonds), and substrate sites (squares), and edges are contact rearrangements between protein residues and protein-ligand site contacts. Two of four symmetry-related contact rearrangement clusters are shown for clarity. Each protein residue is colored according to the color in Figure 2B of the quaternary node to which that residue belongs. (B) Quaternary network of Figure 2B, with each edge colored according to the CRN cluster (if any) to which the corresponding quaternary interface contributes. A red edge indicates that a quaternary interface contributes to more than one CRN cluster. A quaternary interface contributes to a CRN cluster if the CRN cluster contains two or more contact rearrangements that cross the quaternary interface.

In Figure 3, we map the QN onto the tertiary (contact rearrangement) network (CRN) and the CRN onto the QN to assess the relationship of tertiary and quaternary communication in PFK. Figure 3A shows a map of residue-residue contact rearrangements where the residues are colored by rigid-body affiliation, revealing that residues in the CRN clusters of PFK belong to multiple quaternary nodes. This suggests that tertiary communication can link multiple quaternary nodes and can contribute to the interfaces between such nodes. In addition, Figure 3B shows that while some quaternary interfaces (QIs) contribute to no CRN cluster and a few QIs contribute to multiple CRN clusters, most QIs contribute to exactly one of the four CRN clusters. In several cases, a set of 4 to 5 QIs (e.g. the set of green-colored QIs in Figure 3B) contributes to the same CRN cluster, which means that each such set of quaternary motions is interdependent at the residue scale. These graphical representations of PFK motions suggest that tertiary and quaternary communication are interdependent in allosteric proteins.

### Integrating Tertiary and Quaternary Networks into One Model

To capture the interdependence of tertiary and quaternary networks, we modify the QN into a “global communication network” (GCN) representation that incorporates tertiary communication represented by the CRN. To create the GCN from the QN, we first explicitly represent each cluster from the CRN as a single “tertiary node.” Second, we create an edge between any tertiary node and any quaternary node which intersect significantly, as defined by shared residues. These shared residues give rise to interdependence between tertiary and quaternary structural changes.

Occasionally, a small rigid body or a flexible segment in the QN shares most of its residues with a tertiary node, which suggests that that quaternary node is better represented as a part of the tertiary node. Even though most internal contacts in a typical rigid body do not rearrange, a small rigid body will overlap strongly with a tertiary node if most of its residues rearrange contacts with residues from neighboring quaternary nodes. Thus, we “annex” these types of rigid bodies and flexible segments into the appropriate tertiary node rather than define an edge between them. In addition, we add an edge between any tertiary node and any substrate or effector site that is part of the corresponding CRN cluster. Finally, as with the QN, we define the allosteric unit in the GCN as the at least 2-connected subset of the graph. As with a ligand node, a tertiary node forms part of an allosteric unit in the GCN if it intersects with both rigid-body partners of any quaternary motion in the GCN. The details of GCN construction and calculations are given in the methods.

**Figure 4:**
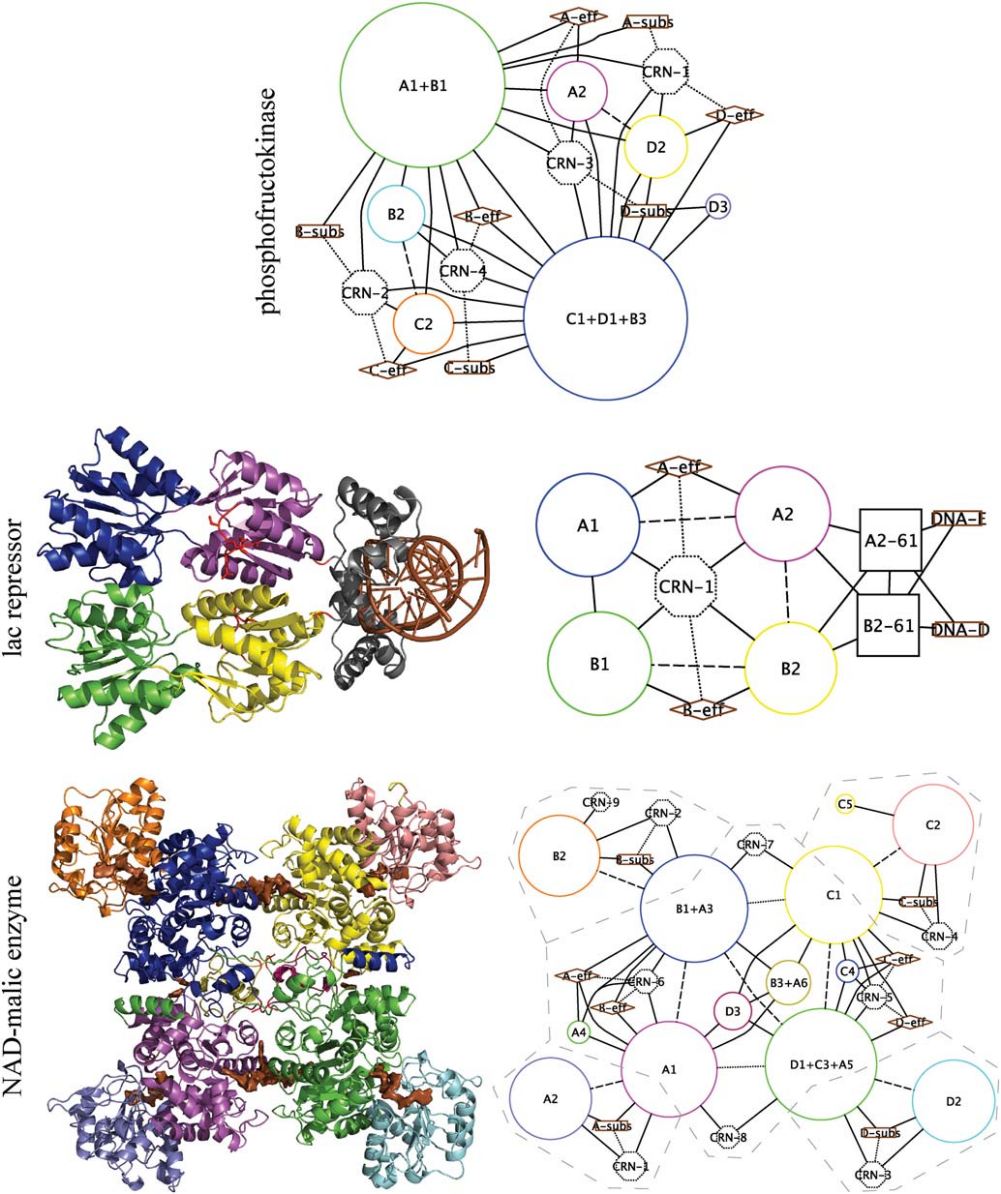
Global communication networks in three proteins. Global communication networks (GCNs) integrate tertiary (contact rearrangement) and quaternary networks. Quaternary nodes, substrate and effector sites, quaternary interfaces, and quaternary node – ligand site interactions are represented as in Figure 2 (for lac repressor (LacR), the DNA molecules are represented as substrates). Each quaternary node is mapped to its position in the three-dimensional structure of the active state (1EFA for LacR and 1PJ2 for malic enzyme) by the node's outline color (see Figure 2 for the mapping for PFK). Tertiary nodes comprising 10 or more residues or contacting a ligand site are represented as octagons with the area proportional to the number of residues; these nodes are numbered by size from largest to smallest. In addition, in lac repressor, square nodes represent segments present only in the active state structure. Modifications to both tertiary and quaternary node areas have been made to account for the participation of some residues in both tertiary and quaternary nodes. Quaternary node-tertiary node edges indicate intersections (shared residues) between these two types of nodes, and an edge between a tertiary node and a ligand site indicates that the ligand site participates in the CRN cluster corresponding to the tertiary node. Furthermore, for malic enzyme, grey dashed lines mark the allosteric unit boundaries (for both PFK and lac repressor, the entire protein is the allosteric unit). Finally, the density of dashing of a quaternary edge is proportional to the interfacial contact rearrangement *f*_CR_. Solid: *f*_CR_<10% (conserved interface); dashed: 10%≤*f*_CR_≤50% (moderately rearranged); dotted: *f*_CR_>50% (extensively rearranged). See the methods for the full details of the GCN representation and associated calculations. Graphs drawn by yEd graph editor. Specific residues comprised by each quaternary node are available in Dataset S1.

Figure 4 shows three examples of GCNs. In the GCN of PFK (top), the tertiary nodes have annexed most of the smaller quaternary nodes in the system: the small rigid bodies A3, B4, C3, and D4 and the flexible segment 156–162 in each subunit. That is, these small quaternary nodes are heavily involved in the tertiary network (CRN) for PFK. Unlike in either the QN or the CRN for PFK, a single allosteric unit of the GCN links all substrates and effectors and includes all tertiary and quaternary nodes, which indicates that the combination of tertiary and quaternary networks is critical for global allosteric communication in this system. In addition, all but two major (i.e. non-annexed) quaternary interfaces undergo rearrangement of less than 10% of the residue-residue contacts across the interface (indicated by line styles of the quaternary edges). That is, most major quaternary motions in PFK do not impinge directly on the tertiary network of the system.

The GCN of the DNA-binding protein lac repressor (LacR, Figure 4 middle) is topologically simpler than that of PFK. LacR includes two quaternary nodes per subunit plus N-terminal DNA-binding domains. A central tertiary node links the two effector sites, and a single allosteric unit comprises the entire GCN and appears to link effector and DNA-binding sites. However, the presence of the DNA-binding domains in only the active state structure hampers the unambiguous identification of effector-DNA connectivity. Finally, all but one of the quaternary interfaces of LacR experience moderate (10–50%) contact rearrangement, which suggests that contact rearrangement is more directly involved in the quaternary transition than in PFK.

In NAD-malic enzyme (Figure 4, bottom), even the global communication network fails to link substrate and effector sites. Grey dashed lines in the figure divide the five allosteric units which are disconnected by the CCH. The central allosteric unit, bounded by quaternary nodes A1, B1+A3, C1, and D3+C3+A5, links the four effectors, two pairs of which are also linked by small tertiary nodes. However, each substrate site is in a separate allosteric unit that is isolated because it is only 1-connected to the central allosteric unit and to the other three substrate sites. To check for obvious mechanochemical allosteric effects our networks may have missed, we visually examined an interpolation between the coordinates of the inactive and active structures of malic enzyme (Video S1) but observed nothing obvious.

### GCNs in the Allosteric Benchmark Set

To expand upon Figure 4, Figure S2 provides an atlas of GCN figures for 25 selected proteins from the allosteric benchmark set [29], including all DNA-binding proteins and enzymes (except those in Figure 4) and several representative examples of signaling proteins. For malic enzyme, PFK, tet repressor (TetR), IRK, ATP sulfurylase, and lactate dehydrogenase, we also provide supplemental movies (Videos S1, S2, S3, S4, S5, S6, respectively) of an interpolation between the inactive and active structures with each rigid body and flexible segment colored according to the rigid-body decomposition. The coordinate interpolation was performed by the multi-chain algorithm of the morph server [34].

In addition to these supplemental figures, we provide the raw data for each of the 51 proteins in the allosteric benchmark set in Dataset S1 and at http://graylab.jhu.edu/allostery. Specifically, for each protein a file lists the residues in each component of the GCN, and a PyMOL script is available to highlight the rigid and flexible segments of the structure. The quaternary network and GCN are available as graph modeling language (GML) files which can be viewed with programs such as yEd (http://www.yworks.com) according to instructions provided.

Figure S2 shows considerable variation in GCN topology over different classes of the allosteric benchmark. G proteins like ras bind a target protein when GTP is bound at the effector site. The ras GCN comprises one rigid body and one tertiary node, and connectivity between effector and “substrate” (target protein ralGDS) in this protein requires only the tertiary network. Response regulators like CheY are activated when a residue in the protein is phosphorylated. The CheY GCN is similar to that of ras; a small tertiary node links the phosphorylation site (mimicked by beryllium fluoride) to a peptide fragment from the target protein FliM. Protein kinase IRK is activated by phosphorylation of the well-known activation loop [35]. The GCN of IRK is more complex than that of ras or CheY; IRK's GCN contains two major quaternary nodes between which there is a domain motion plus the tertiary node CRN-1 that incorporates the activation loop. Representation of this phosphorylation as an effector site attached to CRN-1 suggests that as with ras and CheY, the substrate-effector connection in this protein is reliant primarily upon the tertiary network.

The results for ras, CheY, and IRK suggests that substrate-effector connectivity in signaling proteins relies primarily upon the tertiary network, possibly because of typically short substrate-effector distances in such proteins. By contrast, the GCNs of enzymes and DNA-binding proteins in Figure 4 and Figure S2 are typically larger and contain more quaternary nodes. GCNs of enzymes larger than those in Figure 4 (e.g. ATP sulfurylase and glcN-6-P deaminase) often contain tens of quaternary and/or tertiary nodes linking distant substrate and effector sites. Thus, in DNA-binding proteins and enzymes, it is possible to investigate the relative contributions of tertiary and quaternary networks to long-distance allosteric communication in proteins.

### Substrate-Effector Connectivity in Large Proteins

The allosteric benchmark set contains 8 DNA-binding proteins and 18 enzymes [29]. Unfortunately, substrate-effector connectivity cannot be analyzed in all of these 26 proteins because substantial portions of the substrate and/or effector binding regions are absent in one or more structures. Six DNA-binding proteins (all except met repressor (MetR) and TetR) are excluded because the DNA-binding domain is absent in one or more of the structures, and the enzyme caspase is excluded because most of the substrate site is absent in the inactive structure. While hemoglobin exhibits homotropic connectivity among the four hemes (see Figure S2), it is excluded because it is not heterotropic. For the remaining 18 proteins, Table 1 quantifies substrate-effector connectivity in proteins via the global allosteric transition (represented by the GCN), tertiary structural changes (represented by the CRN) and quaternary structural changes. GCN_Q_, the portion of the QN not annexed into the tertiary network in the construction of the GCN, represents the quaternary structural changes.

**Table 1:** Substrate-effector connectivity in 18 proteins.

Protein Name	% Heterotropic Paths				
	Tertiary Network	Quaternary Network	Global Network	Interdependence	Communication Class
MetR	0%	0%	0%	-	-
TetR	0%	100%	100%	0%	Quaternary
Anthranilate synthase	50%	50%	100%	25%	Mixed
ATP sulfurylase	0%	0%	100%	100%	Interdep
ATP-PRT	0%	0%	100%	100%	Interdep
ATCase	0%	0%	0%	-	-
Chorismate mutase^a^	0%	100%	100%	0%	Quaternary
DAHP synthase	25%	25%	100%	75%	Interdep
FBPase-1	100%	0%	100%	0%	Tertiary
GTP cyclohydrolase I	100%	15%	100%	0%	Tertiary
glcN-6-P deaminase	0%	0%	100%	100%	Interdep
Glycogen phosphorylase	0%	50%	100%	50%	Interdep
Lactate DH	100%	0%	100%	0%	Tertiary
NAD-malic enzyme	0%	0%	0%	-	-
Phosphofructokinase	25%	25%	100%	56%	Interdep
Phosphoglycerate DH	0%	100%	100%	0%	Quaternary
PTP1B	0%	0%	100%	100%	Interdep
Uracil PRT	100%	0%	100%	0%	Tertiary
Hits (≥20% of paths)	7/18	7/18	15/18		4T/3Q/1M/7I
Hits (all paths)	4/18	3/18	15/18		

“Tertiary network” refers to the contact rearrangement network and “quaternary network” refers to the quaternary subgraph of the global communication network (GCN_Q_). Interdependence is defined as the fraction of observed paths in the GCN that were not observed via either the tertiary or the quaternary network. “Communication class” refers to the dominant type of communication in a system, with “mixed” meaning that both tertiary and quaternary communication capture 50% or more of the paths and “interdep” meaning that interdependence is 50% or higher. “Hits” indicates the number of proteins in the set which exhibit connectivity according to two different thresholds. Percent heterotropic paths has been updated from our previous work [12] in a few proteins (see methods for details).

a Substrate binding site determined from a third structure, 4CSM.pdb, with a competitive inhibitor bound at the substrate site.

The maximum number of heterotropic paths in a protein (the number of substrate sites times the number of effector sites) is achieved if a single allosteric unit links all substrate and effector sites. We quantify heterotropic connectivity using the metric *f*_paths_, defined as the observed number of such paths through a given network divided by the maximum. We set two criteria for connectivity: moderate (*f*_paths_≥20%), and stringent (*f*_paths_ = 100%). The moderate criterion is met in 7 proteins by the tertiary network, in 7 proteins by GCN_Q_, and in 15 proteins by the GCN, while the stringent criterion is met in 4 proteins by the tertiary network, in 3 proteins by GCN_Q_, and in 15 proteins by the GCN. Furthermore, in 7 of the 15 systems stringently connected by the GCN, 50% or more of the heterotropic paths via the GCN are “interdependent” paths that do not exist via either the tertiary network or GCN_Q_. That is, many *individual* substrate-effector communication pathways in these systems involve both tertiary and quaternary effects.

Because homotropic (substrate-substrate and effector-effector) connectivity in allosteric proteins might behave differently than heterotropic connectivity, we examine homotropic connectivity in the 18 proteins plus four others in Table S1. Moderate homotropic connectivity is about twice as widespread among these 22 proteins as moderate heterotropic connectivity via either the CRN or GCN_Q_. However, no large difference exists between the homotropic and heterotropic connectivity rates for the CRN at the stringent criterion or for the GCN at either the moderate or the stringent criterion.

### Allosteric Pathways in the GCN

**Figure 5:**
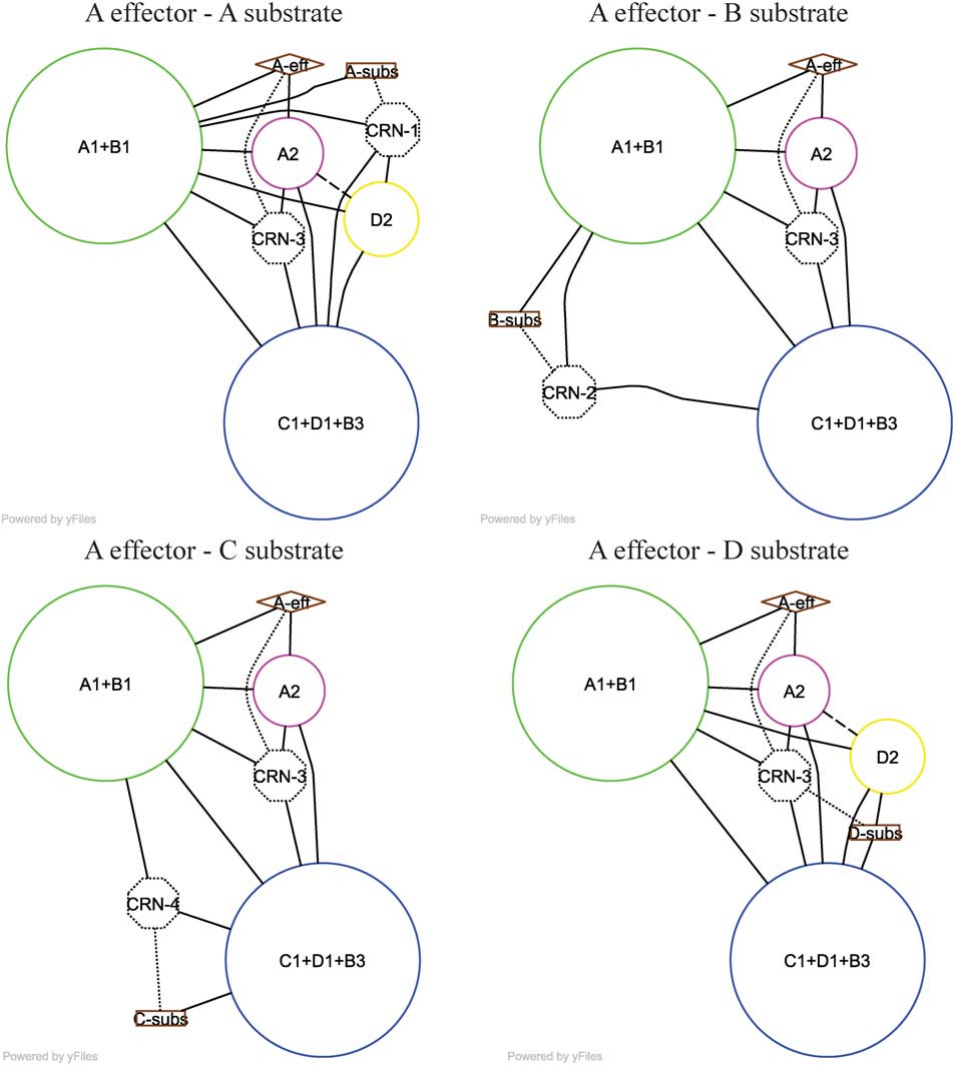
Proposed individual substrate-effector “pathways” in the PFK GCN. The complete GCN of PFK is shown in Figure 4. We define a “pathway” between a substrate and an effector site in the GCN as the shortest loop containing them, plus any cross-interactions among members of that loop. If two or more loops are tied for the shortest, the union of all such loops constitutes the pathway. This pathway is the smallest (by number of nodes) subset of the GCN required to form an allosteric unit containing the two sites. Four symmetrically unique paths emanating from the effector site of chain A are shown.

By extension of the cyclic connectivity hypothesis (CCH), the GCN representation can also reveal substrate-effector “pathways” within an allosteric unit for a protein. Under the CCH, such a pathway must be a cyclically connected subgraph (itself an allosteric unit) rather than a simple linear chain. Specifically, we hypothesize that in the GCN, the “pathway” between any two sites comprises the smallest 2-connected subgraph of the GCN containing the two points. While a systematic analysis of these complex paths over the allosteric benchmark set is beyond the scope of this work, we demonstrate paths in Figure 5 and Figure S3 for the three proteins of Figure 4.

For PFK, Figure 5 shows four symmetrically unique substrate-effector pathways emanating from the effector site of chain A to each of the four substrate sites. Each of these “pathways” contains five to six tertiary and/or quaternary nodes. Three cycles of length 6 (one such cycle is A-eff, A1+B1, A-subs, CRN-1, D2, A2, A-eff) unite to form the minimal subgraph linking the substrate and effector of chain A; this subgraph comprises tertiary nodes CRN-1 and CRN-3 and quaternary nodes A1+B1, A2, C1+D1+B3, and D2. We also include cross-interactions among these nodes (e.g. A1+B1 – C1+D1+B3) in the minimal subgraph. Redundant cycles and cross-interactions may be important for strength of substrate-effector connections over long distances.

The four symmetrically distinct substrate-effector subgraphs in Figure 5 are related in important ways. Nodes A1+B1, C1+D1+B3, A2, and CRN-3 participate in all four of the subgraphs, suggesting that these nodes are important for all of the heterotropic paths involving the effector site of chain A and that mutation of residues at interfaces among these nodes could affect multiple allosteric pathways. Furthermore, all of the “pathways” utilize a substantial portion of the GCN, possibly indicating a high degree of cooperation between different substrate-effector paths in PFK.

Figure S3 shows one pathway apiece for LacR and malic enzyme. In each of the proteins, the pathway shown comprises a substantial part of the protein structure, as the PFK pathways do in Figure 5. In LacR, all four substrate-effector paths are symmetry-related, and the representative pathway between the effector site of chain A and DNA chain E includes the N-terminal and DNA-binding domains but neither of the C-terminal domains. In malic enzyme, the pathway between the effector sites of chain A and D includes most of the residues in the central allosteric unit, but many of the smaller nodes in this unit are absent and thus probably not important for communication between these distant effector sites.

### Statistics of Global Communication Networks

**Figure 6:**
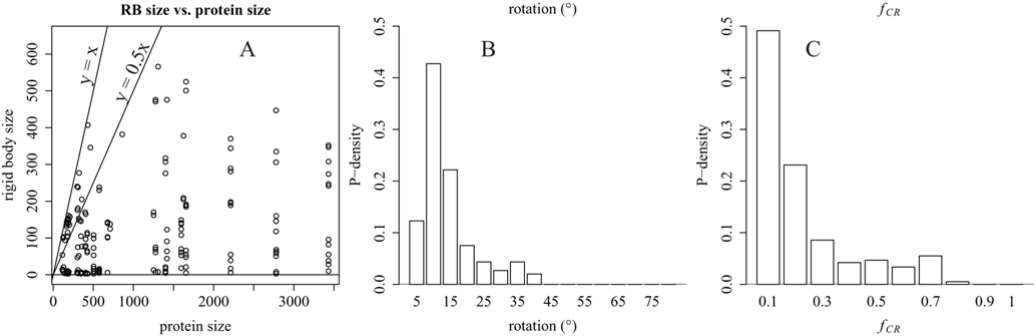
Statistics of quaternary nodes and rigid-body motions in global communication networks. (A) Rigid-body size versus protein size (number of residues) for all rigid bodies in the global communication networks (GCNs) of all proteins in the allosteric benchmark set [29]. A point at (2800, 1200) is excluded for clarity. (B) histogram of rotation angle for all edges between all rigid bodies in these GCNs. (C) histogram of interfacial contact rearrangement *f*_CR_ for all edges between rigid bodies in these GCNs. For constructing the histograms in (B) and (C), each edge in each GCN is weighted by the inverse of the number of asymmetric units in the protein to normalize for multiple symmetry-related motions in some oligomers. Bin labels refer to the upper bound of the bin. Only edges between rigid bodies both of which comprise 30 or more residues are included in the histograms.

While the quaternary and global networks reveal the allosteric communication pathways arising from quaternary motions, the properties of the underlying quaternary motions may also be important for the structural basis of protein allostery. We investigate these properties in Figure 6 through statistical analyses of several key rigid-body motion parameters in the 51 proteins of the allosteric benchmark set [29].

Figure 6A examines the distribution of rigid body sizes. While allosteric proteins comprise up to about 3400 residues, rigid bodies within these proteins comprise fewer than 600 residues in all but one case (a 1211-residue rigid body from the 2780 residue, 20-subunit protein GTP cyclohydrolase), and the range of rigid body sizes varies little with protein size. In addition, rigid bodies commonly comprise up to 100% of the protein in proteins of fewer than 500 residues, and rigid bodies commonly comprise up to half the protein in proteins of fewer than 1500 residues.

Figure 6B shows that most rotations between rigid bodies are less than 20°. The only rotations greater than 40° are the 90° and 81° rotations between the largest domain of the EfTu and two respective smaller domains. Hayward has surveyed domain motions in a set of 24 proteins, most of which are non-allosteric [36]. Most rigid-body rotations in Figure 6B populate the lower range of values observed by Hayward, which suggests that allosteric quaternary motions may be more restricted than protein motions in general.

Figure 6C provides a deeper view of the interfacial contact rearrangement *f*_CR_ examined in Figure 4. The higher *f*_CR_ is for a quaternary motion, the more interdependent that motion is with the tertiary (contact rearrangement) network. For a surprisingly large 49% of the quaternary motions surveyed, fewer than 10% of residue-residue contacts rearrange at the interface; these motions could be described as almost “purely quaternary.” These 49% of quaternary motions likely involve relatively little contact rearrangement because the two rigid bodies have a small rotation relative to each other or most of the contacts between the two rigid bodies lie near the axis of rotation. The remaining 51% of quaternary motions suggest that significant interdependence of quaternary motions with tertiary communication is common but not universal in allosteric proteins.

We also analyzed additional parameters of the rigid-body motions toward identifying significant translational components (data not shown). Ten percent of rigid-body motions surveyed in Figure 6 are “pure rotations” involving translation of the center of mass of the smaller partner of 1 Å or less. For 61% of rigid-body motions, the center of mass of the smaller partner translates significantly, but this translation can be accounted for by an axis of rotation passing near the interface residues between the two rigid bodies. In 18% of rigid-body motions, the axis passes through the interface but there is translation of more than 1 Å parallel to the rotation axis. In 11%, the center of mass of the smaller partner translates significantly, and the axis does not pass close to the interface. That is, about 29% of rigid-body motions in the benchmark set involve significant translational components, while 71% appear to be mostly rotational. Protocol S1 details the calculation of the position of the rotation axis relative to the interface.

## Discussion

The global communication network (GCN) representation integrates both tertiary (residue-scale) and quaternary (domain- and subunit-scale) structural changes, both of which are known to be important to allosteric communication [11]. The observation that the GCN analyzed according to the cyclic connectivity hypothesis (CCH) accounts for substrate-effector connectivity in 83% of proteins surveyed argues for the importance of the GCN representation and the validity of the CCH.

In addition, the substantially higher connectivity rate for the GCN than either the tertiary or the quaternary network argues that different scales of motion are important for allosteric communication in different portions of the protein structure. Most interestingly, tertiary and quaternary scales of motion commonly act interdependently rather than separately toward allosteric coupling.

Previous works have also offered evidence that gross properties of the protein structure can account for protein functions like allostery. For example, dynamics calculated from highly coarse-grained (i.e. domain-scale) elastic networks match closely the dynamics calculated from residue-scale elastic networks [20],[37].

Our derivation of pathways of allosteric coupling from the gross topology of tertiary and quaternary structural changes builds upon the MWC and KNF models. The MWC model [1] emphasizes the conservation of symmetry at the quaternary structure level for driving cooperative transitions between different allosteric structural regimes. While our topology-based model does not require symmetry for coupling, symmetric topology could restrict the range of accessible conformations and thereby enhance coupling within an allosteric unit of a global communication network. In addition, in the KNF model [2], tertiary structural changes propagate a ligand-binding signal from the interior of a subunit to a quaternary interface, where it would induce an equivalent change in an adjacent subunit. From a network perspective, we have observed two additional roles of tertiary changes: to directly link substrate and effector sites [12] and to couple together at the residue scale what appear at the quaternary scale to be independent motions.

In addition to incorporating changes in the ensemble-average (crystal) structure as did the MWC and KNF models, a comprehensive theory of allostery must incorporate changes in protein flexibility, that is, in conformational entropy [38]. However, the strong success of the GCN argues that allosteric mechanisms reliant primarily upon observable changes in the ensemble-average structure are common and may be dominant.

For proteins not connected via GCNs based upon comparing end-state structures, the theory of the GCN could be extended to networks of dynamic changes. By incorporating information about the dynamic perturbations associated with the allosteric transition determined by solution experiments (e.g. [7],[39],[40]) or computational methods (see the following paragraph), the GCN might account for allosteric communication in more proteins than the 83% achieved in this work.

For example, as a first step toward predicting a GCN for a protein with only one known structure, rigid and flexible substructures predicted from the network of contact and hydrogen bond constraints [26] could be represented as a quaternary network,. In addition, the components of the GCN might be predicted from a single structure via normal mode analysis (NMA) [25],[41],[42]. Predicted rigid bodies could be extracted from an NMA correlation matrix [43], and tertiary nodes of the system could be inferred from the “hinge” regions [44] whose dynamics are highly correlated to the dynamics throughout the protein (such hinge regions overlap strongly with the CRN in myosin [12]).

As a parsimonious representation of the topology of the allosteric transition, the GCN may be useful to guide experiments and computations probing allosteric function. Specifically, the quaternary interfaces in the GCN (especially those which participate in many individual substrate-effector “pathways” in multimeric proteins) are probably important for a range of allosteric functions. Thus, these interfaces are likely important regions of the protein to probe in such approaches. For example, mutational perturbations of residues at globally important interfaces may capture intermediates along the kinetic pathway between inactive and active structural regimes; this could aid works like those of Ackers [13],[14] which capture microstate binding constants of the system. In addition, measurement of the dynamic properties of the quaternary interfaces in the GCNs could help to quantify the balance of enthalpic and entropic allosteric effects. Furthermore, especially for large allosteric proteins, the GCN representation may simplify atomistic simulations and energy landscape computations by limiting the simulation to a relatively small number of biologically relevant degrees of freedom while constraining the internal structure of rigid regions.

Finally, using screening techniques, considerable progress has been made in the discovery of novel allosteric regulation in proteins not previously believed to be allosterically regulated [45]–[48] as well as in the design of switching proteins [49]. A non-allosteric protein whose structure already has a 2-connected topology of domain and/or subunit interfaces may be primed for rational design of allostery. For each quaternary interface in such a protein, mutational perturbation of the interface could increase the sensitivity of the structure of that interface to ligand-binding. Thus, by clarifying the topology of motions required for allosteric communication, our theory of allosteric coupling could guide design of allostery by rational means and/or by targeted random variation.

## Methods

### Rigid-Body Calculations

**Figure 7:**
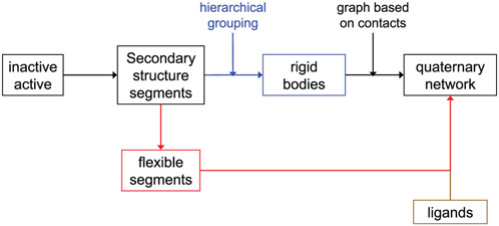
Elucidating quaternary motion network from two protein structures.

Figure 7 outlines the algorithm for elucidating quaternary motions and a network thereof from a comparison of two allosteric protein structures. The steps of this algorithm are detailed in following subsections.

#### Superpositions

All superpositions of protein fragments are based on the C_α_ atoms using the SVDSuperimposer package of BioPython (http://www.biopython.org), which calculates the optimal translation vector and rotation matrix of a set of points using a singular value decomposition algorithm. Furthermore, all superpositions of fragments of 50 or more residues utilize our previously published flexible protein superposition algorithm [29]. All rmsds of groups of residues are based on the C_α_ atoms unless otherwise stated.

#### Breakdown into secondary structure fragments

For each chain in the protein, clustalw [50] identifies sequentially equivalent fragments between the two structures as well as fragments unique to one state. DSSP [51] calculates the secondary structure for the inactive and active state conformations of the chain. We define a consensus secondary structure for the aligned fragment as helix or loop for residues with conserved helical (α, 3_10_, or π) or β-strand (extended) structure, respectively, and loop for all others. We then partition each sequentially contiguous fragment into consensus secondary structure fragments and adjust fragment boundaries as needed so that each fragment is no less than 3 and no more than 10 residues long. For this adjustment, we first join any fragment shorter than 3 residues to the preceding fragment (if the N-terminal fragment is shorter than 3 residues, we join it to the following fragment). Then we subdivide all resulting fragments longer than 10 residues as evenly as possible into the minimum number of fragments shorter than 10 residues.

#### Net rigid-body motion between two fragments

To account for crystallographic uncertainty when identifying a motion between two rigid bodies, we subtract the internal motions of the rigid bodies as two previous algorithms have done [30],[31]. For two fragments of a protein, we define
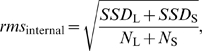
where *SSD* of a fragment is the sum of square displacements over all C_α_ atoms after superposition, *N* is the size in number of residues, and the subscripts S and L refer to the small and large protein fragments, respectively. To calculate the gross rigid-body motion of S relative to L, *rms*_RB_, we first determine the optimal rotation matrix **R_L_** and translation vector **T_L_** to superimpose *L*_B_ onto *L*_A_, where A and B are the two conformations of the protein. We then transform *S*_B_ into the reference frame of *L*_A_ using **R_L_** and **T_L_** to produce 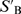 and calculate *rms*_RB_, the total rigid-body motion, between *S*_A_ and 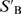. We choose L as the reference frame for *rms*_RB_ to minimize the distortion of *rms*_RB_ by lever-arm effects away from the interface between L and S, by analogy to the use of the larger docking partner as the reference frame to quantify protein docking model error [52]. Finally, we calculate the net rigid-body motion metric, *rms*_net_ = *rms*_RB_−*rms*_internal_.

#### Hierarchical grouping of rigid fragments

Hierarchical clustering of subsets of a protein structure based on rigid-body motion parameters has been recognized as a useful way of identifying groups that move collectively in a protein structure [30]–[32], and we use a similar hierarchical grouping approach in this work. Specifically, the leaves of the clustering tree are rigid (local rmsd≤0.8 Å) secondary structure fragments, and the distance metric of clustering is *rms*_net_. The 0.8 Å cutoff is chosen to be slightly less than 1.0 Å, the typical rmsd over the entire protein between two independently solved crystal structures [53]. At the first step, the two leaves with the lowest *rms*_net_ are joined to form a tree representing a larger substructure, and as in standard hierarchical clustering, this procedure repeats until all rigid fragments in the protein are grouped into a single tree. However, each time a new tree is created, that tree's *rms*_net_ relative to any other tree is re-calculated directly rather than by averaging the *rms*_net_ of its elements relative to the other tree. Furthermore, to prevent the formation of physically noncontiguous clusters, we join two groups only if they make at least one atomic contact and one median radius contact.

We define the median radius of a residue as the median distance of its atoms from its centroid. A median radius contact between two residues exists if the two centroids are separated by no more than the sum of their median radii plus 4.0 Å.

Then, we partition the final clustering tree of protein fragments using two criteria. First, we divide a fragment if *rms*_net_ between its two constituent fragments is no less than some cutoff (0.8 Å in most proteins; exceptions noted in Table S2). Second, to enforce the internal rigidity of each rigid body, we divide a fragment if its rmsd superimposed as a unit (*rms*_unit_) is more than the *rms*_net_ cutoff plus 0.2 Å. In principle, any nonzero *rms*_net_ should be significant because internal motions are subtracted, but in each protein, we sought the lowest *rms*_net_ cutoff that would avoid dividing what visually appeared to be structural domains. In addition, in some cases we used cutoffs lower than 0.8 Å to improve the symmetry of rigid-body partitioning between monomers in oligomeric proteins.

To improve the rigor of the algorithm, two other modifications to standard hierarchical clustering are used. First, in multimeric proteins, to avoid disrupting structural domains by the formation of multi-chain clusters in the early stages of clustering, we cluster and partition the rigid fragments within each chain before clustering between chains. Second, to reduce uncertainty in the clustering procedure arising from local conformational change of the leaves, the most locally rigid (local rmsd≤0.5 Å) fragments are clustered first, and moderately flexible fragments (0.5 Å<local rmsd≤0.8 Å) are clustered in only after the rest of the protein has been clustered and partitioned. To determine the flexible segments of the protein, we find all sequentially contiguous chains of highly flexible fragments (local rmsd>0.8 Å).

#### Rigid-body rotation parameters

To calculate the rotational displacement of the smaller partner S relative to the larger partner L, we superimpose 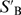 onto *S*_A_ to obtain the optimal rotation matrix 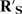 and translation vector t of the center of mass. The rigid-body rotation angle *θ* is determined by
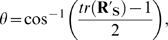
and the rotational displacement axis **u** is calculated as the eigenvector of 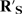 corresponding to the eigenvalue 1. As necessary, we reverse the direction of **u** for consistency with a counterclockwise rotation from conformation A to conformation B.

### Global Communication Network

#### Contact rearrangement networks

Contact rearrangement networks are as previously published [12], except that we have updated the calculation to properly include amino-acid substrate and effector ligands (e.g. trp, phe). The updated CRN calculation revealed previously unobserved heterotropic connectivity for anthranilate synthase and DAHP synthase. The updated GML files are available on our website (http://graylab.jhu.edu/allostery/networks) for these two proteins and ATP-phosphoribosyltransferase, GTP cyclohydrolase, and phosphoglycerate DH, which also contained amino acid substrates and effectors but for which heterotropic connectivity results did not change.

#### Tertiary node-quaternary node edges

In the global communication network (GCN), let any tertiary node represent the set of residues *T_i_* and any quaternary node represent the set of residues *Q_j_*. Let *O_ij_* (overlap) be the cardinality of their intersection:
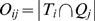
Furthermore, let the tertiary and quaternary fractional overlaps, respectively, be
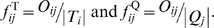
Then, define an edge between *T_i_* and *Q_j_* if *O_ij_*≥5 residues. To deal with small quaternary and tertiary nodes, respectively, for which a small *O_ij_* may be significant, also define such an edge if 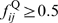 or 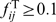, with the exception of annexed nodes as developed below.

#### Annexation of certain quaternary nodes

If *Q_j_* shares most of its residues with *T_i_*, then 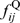 will be large. Such a *Q_j_* is thus participating heavily in tertiary communication, and thus, we join it with *T_i_*. Specifically, if 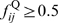 for *Q_j_* of 20 or fewer residues or 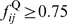 for all other *Q_j_*, we remove *Q_j_* from the GCN and “annex” it into *T_i_*:
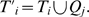
In addition, any edge of such a *Q_j_* with any other quaternary node *Q_k_* is replaced in the GCN by an edge between 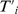 and *Q_k_*. In the limit of 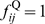, such annexation reduces to a simple deletion of *Q_j_* and all of its edges from the GCN.

Modifications to both tertiary and quaternary node areas have been made to account for the participation of some residues in both tertiary and quaternary nodes. The area of a quaternary node in the GCN is proportional to the number of “core” residues not part of any tertiary node plus half the number of residues that also participate in any tertiary node. Similarly, the area of a tertiary node is proportional to the number of residues annexed from quaternary nodes plus half the number of residues from non-annexed quaternary nodes.

#### Quaternary interfacial contact rearrangement

We quantify contact rearrangement between any two quaternary nodes in the GCN by the fractional contact rearrangement *f*_CR_, which is the number of residue-residue contacts between the nodes with contact rearrangement factor *R*(*i*,*j*)≥0.30 as defined previously [12] divided by the total number of contacts between those two nodes.

#### Substrate-effector connectivity

The fraction of possible heterotropic paths *f*_paths_ in an allosteric protein is
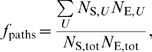
where the sum is over all allosteric units in a given network model of a protein, *N*_S,*U*_ and *N*_E,*U*_ are the respective numbers of substrate and effector sites in a given allosteric unit *U*, and *N*_S,*tot*_ and *N*_E,*tot*_ are the corresponding numbers for the entire protein.

## Supporting Information

Dataset S1 This set contains 51 directories, one for each protein in the allostery benchmark. Within a protein's directory, there are four files: (1) A pymol script (.pml extension) highlighting the rigid bodies and flexible segments. The selection name for each rigid body or flexible segment indicates its position in the quaternary network (QN). Just open the pdb file for either structure of the allosteric protein (the inactive pdb is the first pdb code in the pymol script file name and the active pdb is the second) and run the pymol script. (2) the QN GML - graph modeling language (GML) format of the QN for the protein. In the README file in the top-level directory are instructions for laying out these graphs with the freely available program yEd (http://www.yworks.com). (3) the QN residue lists - chain identifiers and residue ranges for each rigid body and flexible segment in the QN and GCN. (4) the GCN GML. Lay out these GML files according to the instructions in the README.
(0.16 MB ZIP)

Figure S1 A 1-connected cyclic QN. This figure shows a 1-connected cyclic graph where R3 is a cut, that is, a node which disconnects the graph if removed. By the cyclic coupling hypothesis, this graph has two allosteric units: R1-R2-R3 and R3-R4-R5. All motion within R1-R2-R3 can occur if R3-R4-R5 is held rigid as a unit, though steric constraints may give rise to limited coupling between the motions in the two respective allosteric units.
(0.02 MB PDF)

Figure S2 Global communication networks for 25 additional proteins. Global communication networks (GCNs) integrate tertiary (contact rearrangement) and quaternary networks. Quaternary nodes, substrate and effector sites, quaternary interfaces, and quaternary node - ligand site interactions are represented as in figure 2 of the main text. Each quaternary node is mapped to its position in the three-dimensional structure of the active state by the node's outline color. Square nodes represent segments present only in the active state structure. Tertiary nodes comprising 10 or more residues or contacting a ligand site are represented as octagons with the area proportional to the number of residues; these nodes are numbered by size from large to small. Modifications to both tertiary and quaternary node areas have been made to account for the participation of some residues in both tertiary and quaternary nodes. Quaternary node-tertiary node edges indicate intersections (shared residues) between these two types of nodes, and an edge between a tertiary node and a ligand site indicates that the ligand site participates in the CRN cluster corresponding to the tertiary node. Finally, the density of dashing of a quaternary edge is proportional to the interfacial contact rearrangement f_CR_. Solid: f_CR_<10% (conserved interface); dashed: 10%≤f_CR_≤50% (moderately rearranged); dotted: f_CR_>50% (extensively rearranged). See the methods for the full details of the GCN representation and associated calculations. Graphs drawn by yEd graph editor (http://www.yworks.com). Specific residues comprised by each quaternary node are available in the supplemental data sets. For the GCN of ras, ralGDS is connected to rigid-body cluster A1 and tertiary node CRN-1 because residues from both of those nodes bind ralGDS in 1LFD.pdb. Similarly or the GCN of CheY, a peptide fragment of FliM is connected to rigid-body clusters A1 and A2 and tertiary node CRN-1 because residues from both of those nodes bind the FliM fragment in 1F4V.pdb. Continued on the following 9 pages until the mark “End Figure S2.”
(7.84 MB PDF)

Figure S3 Proposed pathways in the GCNs of lac repressor and NAD-malic enzyme. The complete GCNs of these two proteins are shown in figure 4 of the main text, and pathways are calculated as in figure 5 of the main text. One path is shown per protein. A pathway connecting two effectors is shown for malic enzyme because there are no substrate-effector pathways in this protein.
(0.04 MB PDF)

Protocol S1 This file contains the supplementary methods referenced in the manuscript.
(0.10 MB PDF)

Table S1 Homotropic and heterotropic connectivity in 22 proteins. “Tertiary network” refers to the contact rearrangement network and “quaternary network” refers to the quaternary subgraph of the global communication network (GCN_Q_). “Homo” refers to substrate-substrate and effector-effector paths, and “hetero” refers to substrate-effector paths.
(0.05 MB PDF)

Table S2 Proteins with *rms*_net_ cutoffs other than 0.8 Å.
(0.01 MB PDF)

Video S1 Malic enzyme movie: animation of an interpolation between the inactive (1QR6) and active (1PJ2) structures colored by identified rigid bodies, except red, which marks flexible segments. The coordinate interpolation was performed by the multi-chain morph algorithm of the morph server, and the movie was rendered with PyMol.
(1.24 MB MPG)

Video S2 Phosphofructokinase movie: animation of an interpolation between the inactive (6PFK) and active (4PFK) structures colored by identified rigid bodies, except red, which marks flexible segments. The coordinate interpolation was performed by the multi-chain morph algorithm of the morph server, and the movie was rendered with PyMol.
(1.10 MB MPG)

Video S3 Tetracycline repressor movie: animation of an interpolation between the inactive (2TRT) and active (1QPI) structures colored by identified rigid bodies, except red, which marks flexible segments. The coordinate interpolation was performed by the multi-chain morph algorithm of the morph server, and the movie was rendered with PyMol.
(0.94 MB MPG)

Video S4 Insulin receptor kinase (IRK) movie: animation of an interpolation between the inactive (1IRK) and active (1IR3) structures colored by identified rigid bodies, except red, which marks flexible segments. The coordinate interpolation was performed by the multi-chain morph algorithm of the morph server, and the movie was rendered with PyMol.
(0.68 MB MPG)

Video S5 ATP sulfurylase movie: animation of an interpolation between the inactive (1M8P) and active (1I2D) structures colored by identified rigid bodies, except red, which marks flexible segments. The coordinate interpolation was performed by the multi-chain morph algorithm of the morph server, and the movie was rendered with PyMol.
(1.64 MB MPG)

Video S6 Lactate dehydrogenase movie: animation of an interpolation between the inactive (1LTH chain T) and active (1LTH chain R) structures colored by identified rigid bodies, except red, which marks flexible segments. The coordinate interpolation was performed by the multi-chain morph algorithm of the morph server, and the movie was rendered with PyMol.
(1.42 MB MPG)
