# Supplementary material for: Allosteric Communication Occurs via Networks of Tertiary and Quaternary Motions in Proteins
Source: PLoS Comput Biol. 2009 Feb 20;5(2):e1000293. doi: 10.1371/journal.pcbi.1000293 (PMC2634971; doi:10.1371/journal.pcbi.1000293)
Supplement: Figure S1 — A 1-connected cyclic QN. This figure shows a 1-connected cyclic graph where R3 is a cut, that is, a node which disconnects the graph if removed. By the cyclic coupling hypothesis, this graph has two allosteric units: R1-R2-R3 and R3-R4-R5. All motion within R1-R2-R3 can occur if R3-R4-R5 is held rigid as a unit, though steric constraints may give rise to limited coupling between the motions in the two respective allosteric units. (0.02 MB PDF) [file pcbi.1000293.s002.pdf]

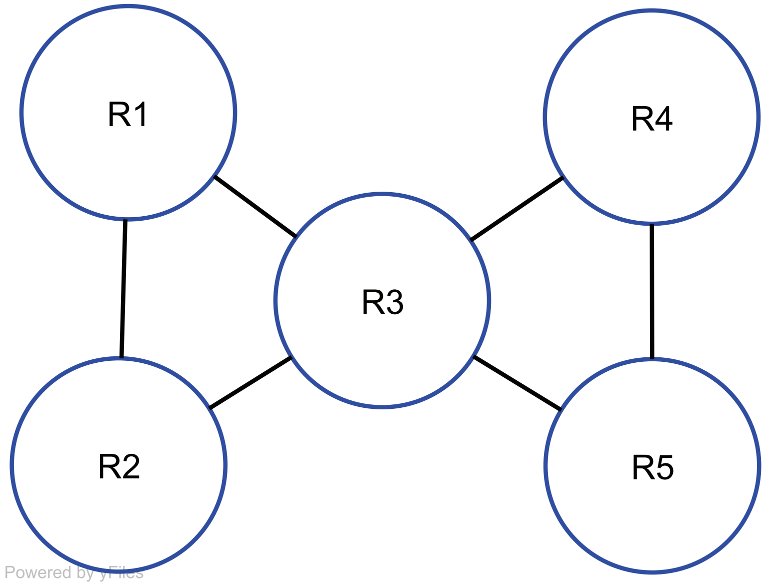

Figure S1: a 1-connected cyclic QN
Supplementary figure 1 shows a 1-connected cyclic graph where R3 is a cut, that is, a node which disconnects the graph if removed. By the cyclic coupling hypothesis, this graph has two allosteric units: R1-R2-R3 and R3-R4-R5. All motion within R1-R2-R3 can occur if R3-R4-R5 is held rigid as a unit, though steric constraints may give rise to limited coupling between the motions in the two respective allosteric units.
