# Supplementary material for: Allosteric Communication Occurs via Networks of Tertiary and Quaternary Motions in Proteins
Source: PLoS Comput Biol. 2009 Feb 20;5(2):e1000293. doi: 10.1371/journal.pcbi.1000293 (PMC2634971; doi:10.1371/journal.pcbi.1000293)
Supplement: Figure S2 — Global communication networks for 25 additional proteins. Global communication networks (GCNs) integrate tertiary (contact rearrangement) and quaternary networks. Quaternary nodes, substrate and effector sites, quaternary interfaces, and quaternary node - ligand site interactions are represented as in figure 2 of the main text. Each quaternary node is mapped to its position in the three-dimensional structure of the active state by the node's outline color. Square nodes represent segments present only in the active state structure. Tertiary nodes comprising 10 or more residues or contacting a ligand site are represented as octagons with the area proportional to the number of residues; these nodes are numbered by size from large to small. Modifications to both tertiary and quaternary node areas have been made to account for the participation of some residues in both tertiary and quaternary nodes. Quaternary node-tertiary node edges indicate intersections (shared residues) between these two types of nodes, and an edge between a tertiary node and a ligand site indicates that the ligand site participates in the CRN cluster corresponding to the tertiary node. Finally, the density of dashing of a quaternary edge is proportional to the interfacial contact rearrangement fCR. Solid: fCR<10% (conserved interface); dashed: 10%≤fCR≤50% (moderately rearranged); dotted: fCR>50% (extensively rearranged). See the methods for the full details of the GCN representation and associated calculations. Graphs drawn by yEd graph editor (http://www.yworks.com). Specific residues comprised by each quaternary node are available in the supplemental data sets. For the GCN of ras, ralGDS is connected to rigid-body cluster A1 and tertiary node CRN-1 because residues from both of those nodes bind ralGDS in 1LFD.pdb. Similarly or the GCN of CheY, a peptide fragment of FliM is connected to rigid-body clusters A1 and A2 and tertiary node CRN-1 because residues from both of those nodes bind the FliM fr [file pcbi.1000293.s003.pdf]

Figure S2: Global communication networks for 25 additional proteins

Global communication networks (GCNs) integrate tertiary (contact rearrangement) and quaternary networks. Quaternary nodes, substrate and effector sites, quaternary interfaces, and quaternary node – ligand site interactions are represented as in figure 2 of the main text. Each quaternary node is mapped to its position in the three-dimensional structure of the active state by the node's outline color. Square nodes represent segments present only in the active state structure. Tertiary nodes comprising 10 or more residues or contacting a ligand site are represented as octagons with the area proportional to the number of residues; these nodes are numbered by size from large to small. Modifications to both tertiary and quaternary node areas have been made to account for the participation of some residues in both tertiary and quaternary nodes. Quaternary node-tertiary node edges indicate intersections (shared residues) between these two types of nodes, and an edge between a tertiary node and a ligand site indicates that the ligand site participates in the CRN cluster corresponding to the tertiary node. Finally, the density of dashing of a quaternary edge is proportional to the interfacial contact rearrangement f_{CR}. Solid: $f_{CR} < 10\%$ (conserved interface); dashed: $10\% \le f_{CR} \le 50\%$ (moderately rearranged); dotted: $f_{CR} > 50\%$ (extensively rearranged). See the methods for the full details of the GCN representation and associated calculations. Graphs drawn by yEd graph editor (www.yworks.com). Specific residues comprised by each quaternary node are available in the supplemental data sets. For the GCN of ras, ralGDS is connected to rigid-body cluster A1 and tertiary node CRN-1 because residues from both of those nodes bind ralGDS in 1LFD.pdb. Similarly or the GCN of CheY, a peptide fragment of FliM is connected to rigid-body clusters A1 and A2 and tertiary node CRN-1 because residues from both of those nodes bind the FliM fragment in 1F4V.pdb.

Continued on the following 9 pages until the mark "End Figure S2."

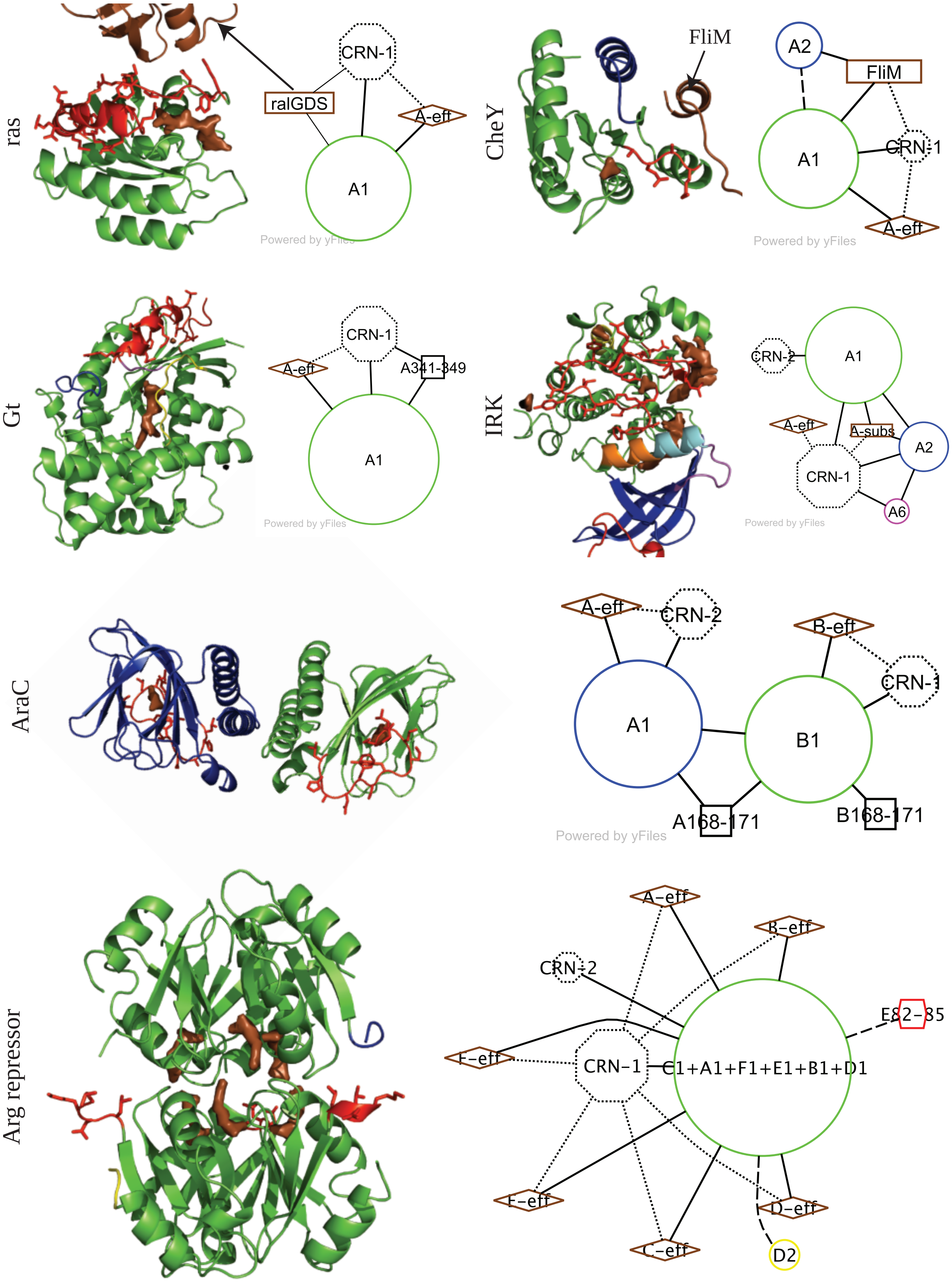
ras
ralGDS
CRN-1
A-eff
A1
CheY
FliM
A2
FliM
A1
CRN-1
A-eff
Gt
CRN-1
A-eff
A341-349
A1
IRK
CRN-2
A1
A-eff
A-subs
A2
CRN-1
A6
AraC
A-eff
CRN-2
B-eff
CRN-1
A1
B1
A168-171
B168-171
Arg repressor
A-eff
B-eff
CRN-2
E82-85
F-eff
CRN-1
C1+A1+F1+E1+B1+D1
E-eff
D-eff
C-eff
D2

Met repressor

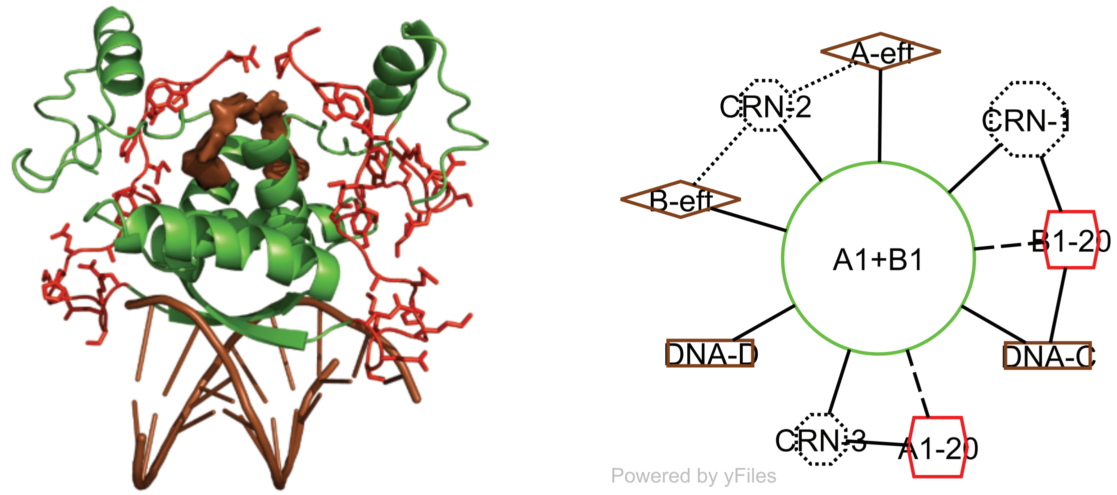

Purine repressor

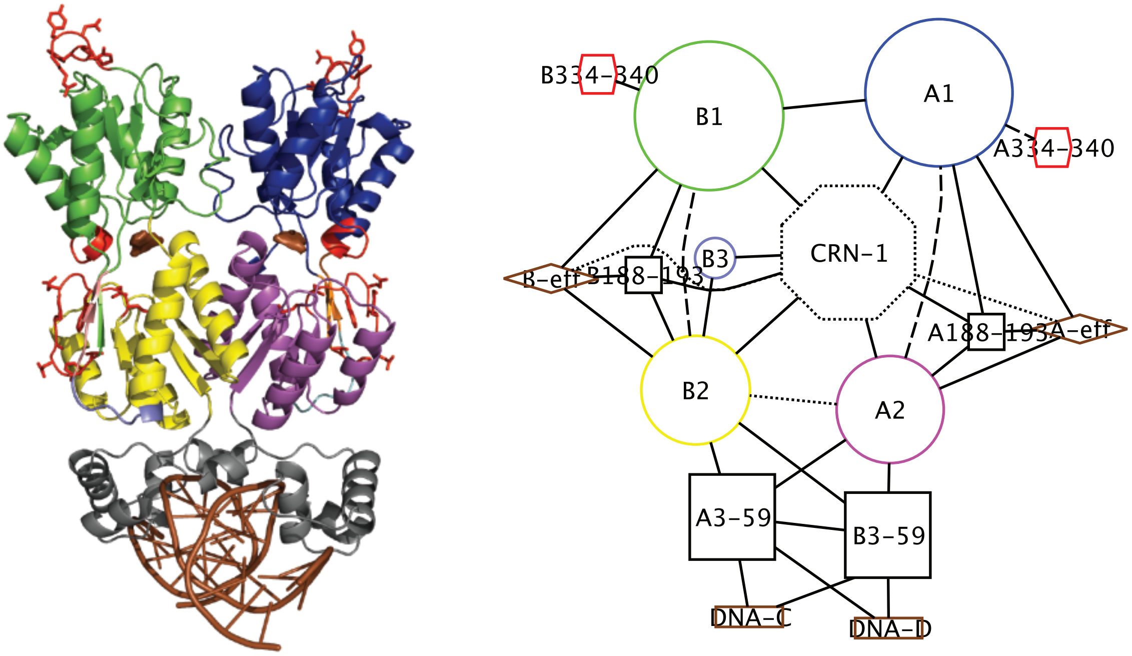

Tet repressor

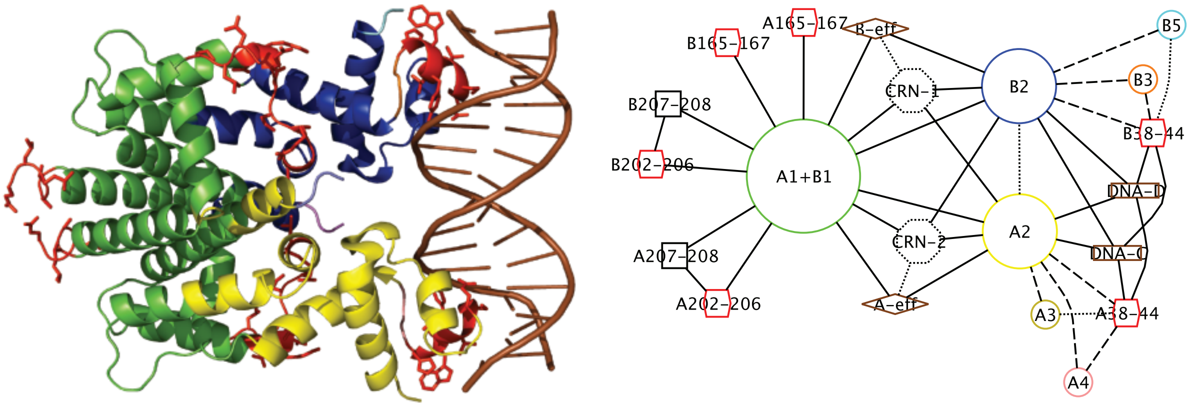

anthranilate synthase

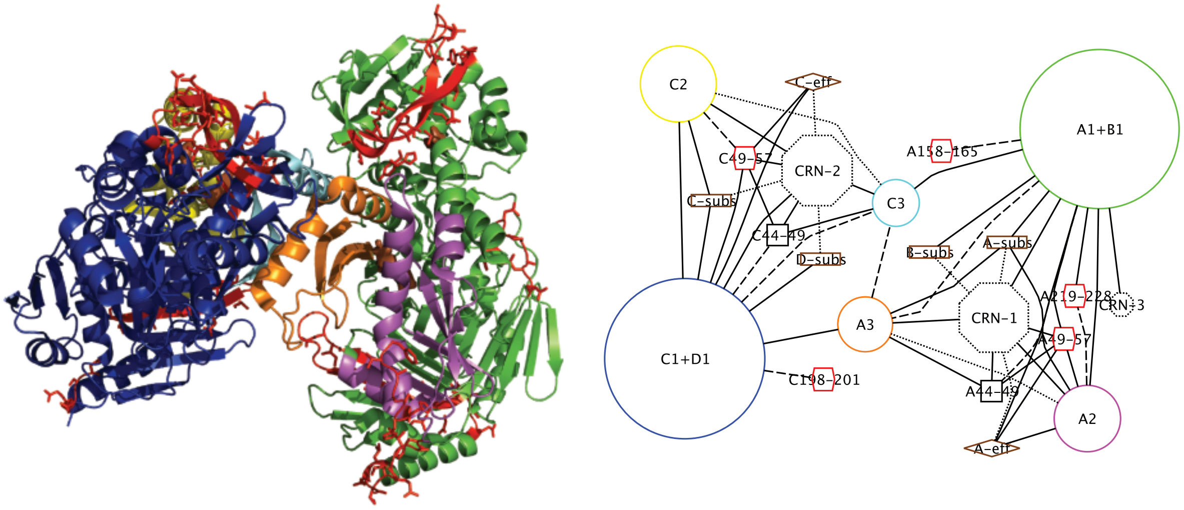

ATP sulfurylase

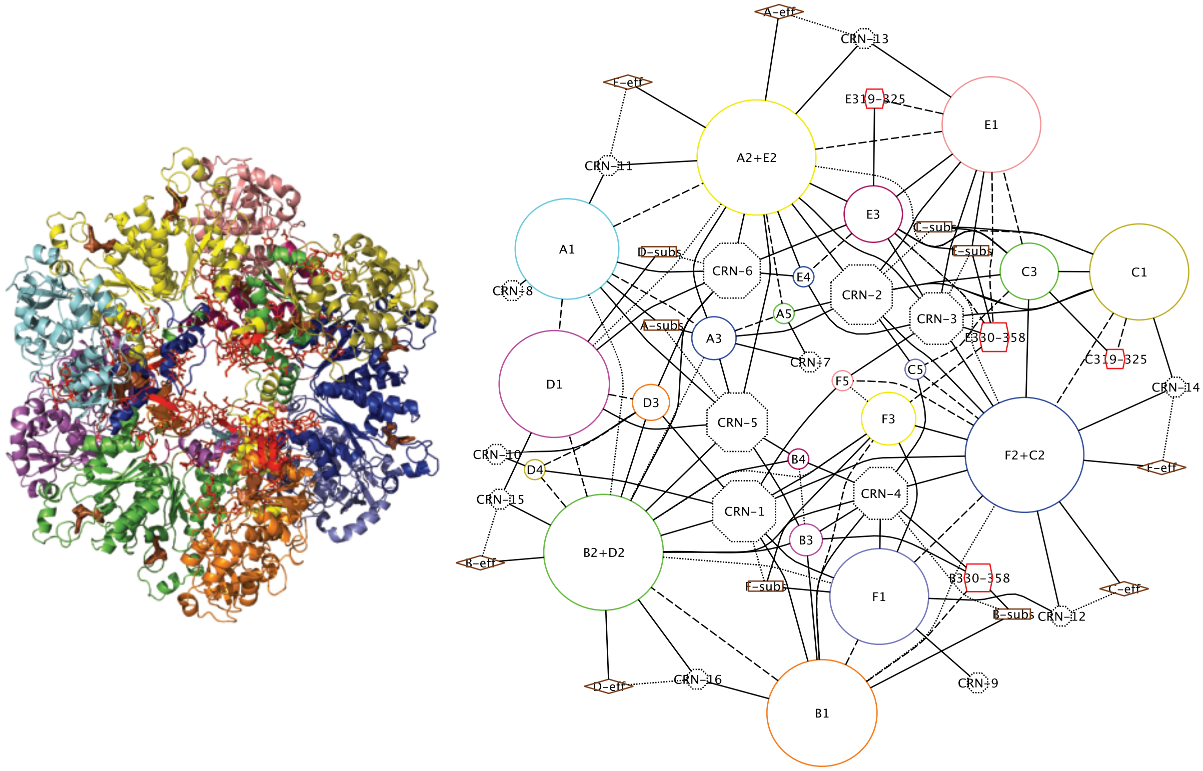

ATP -PRT

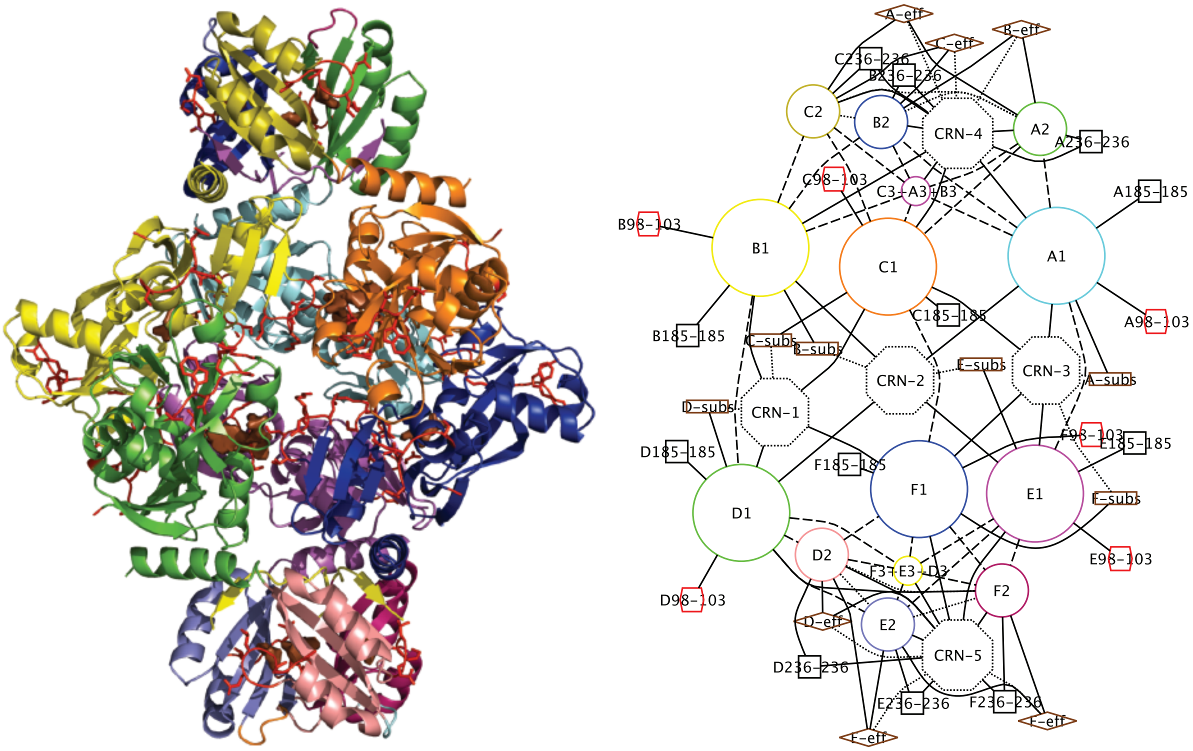

ATCase

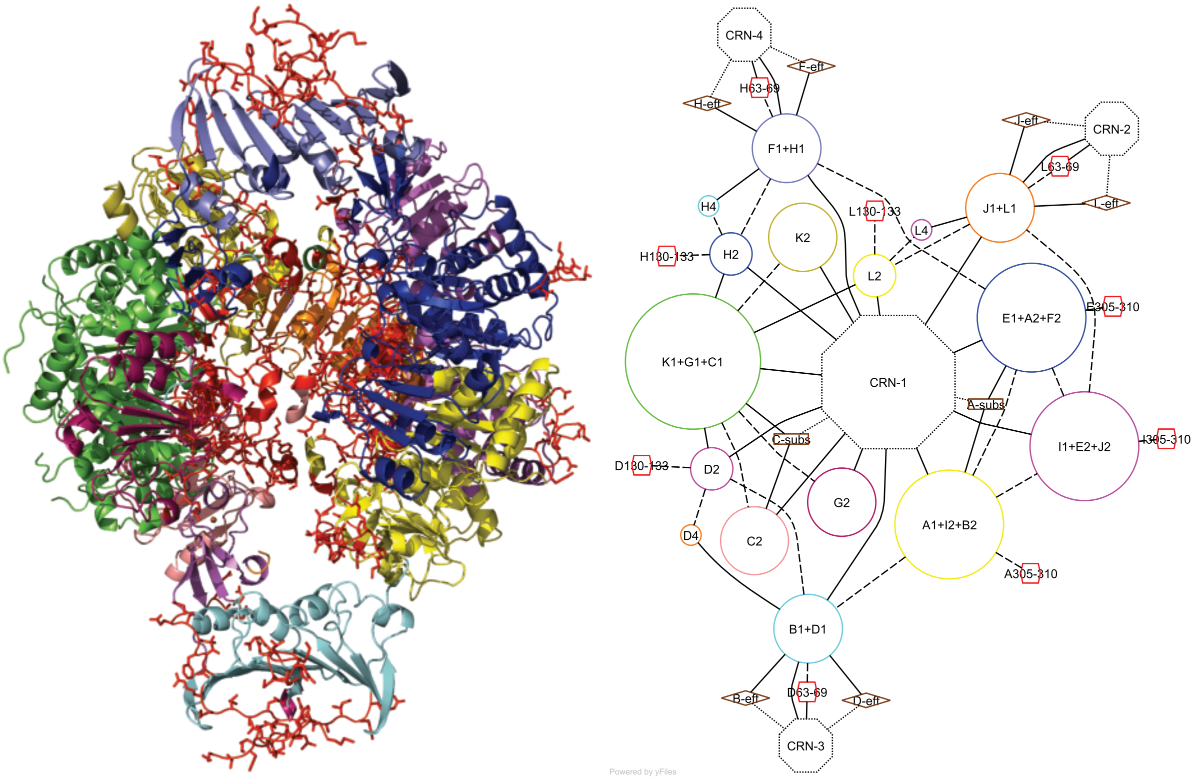

caspase

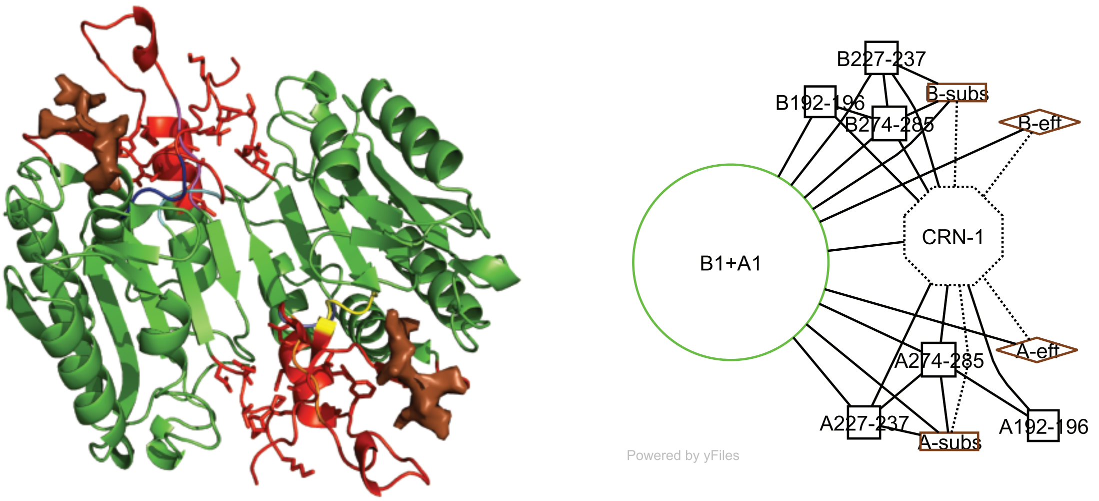

chorismate mutase

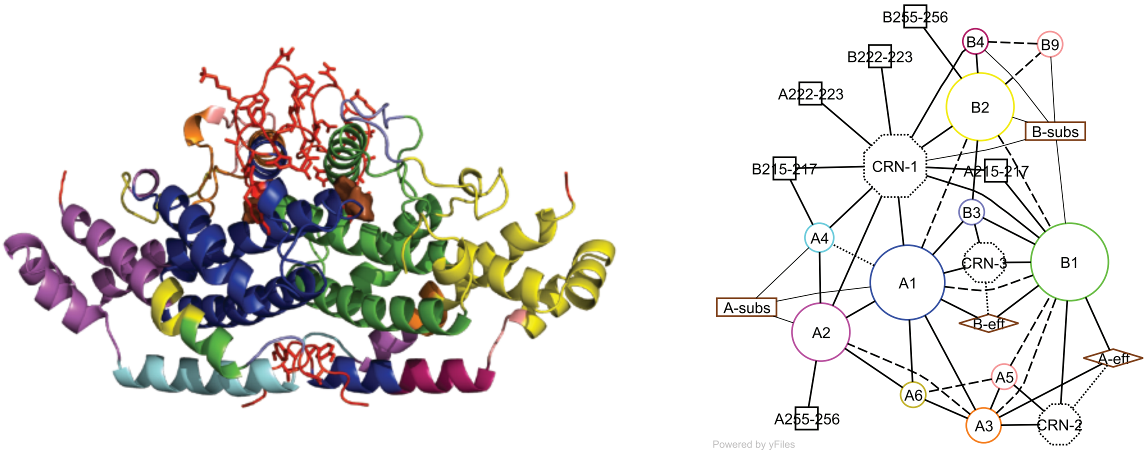

DAHP synthase

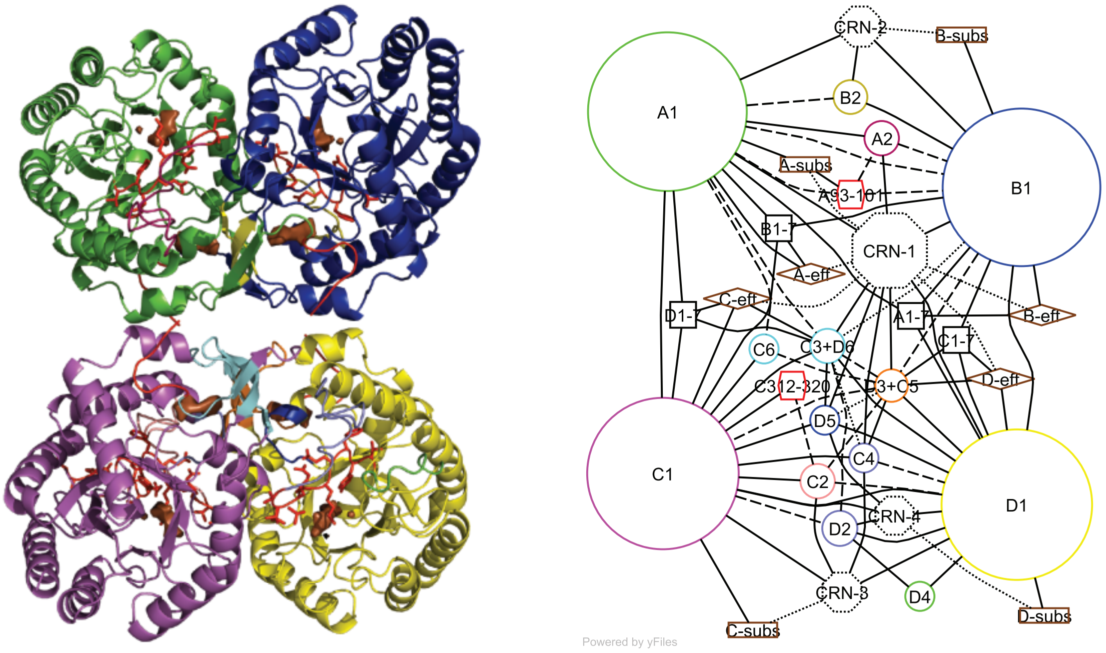

FBPase-1

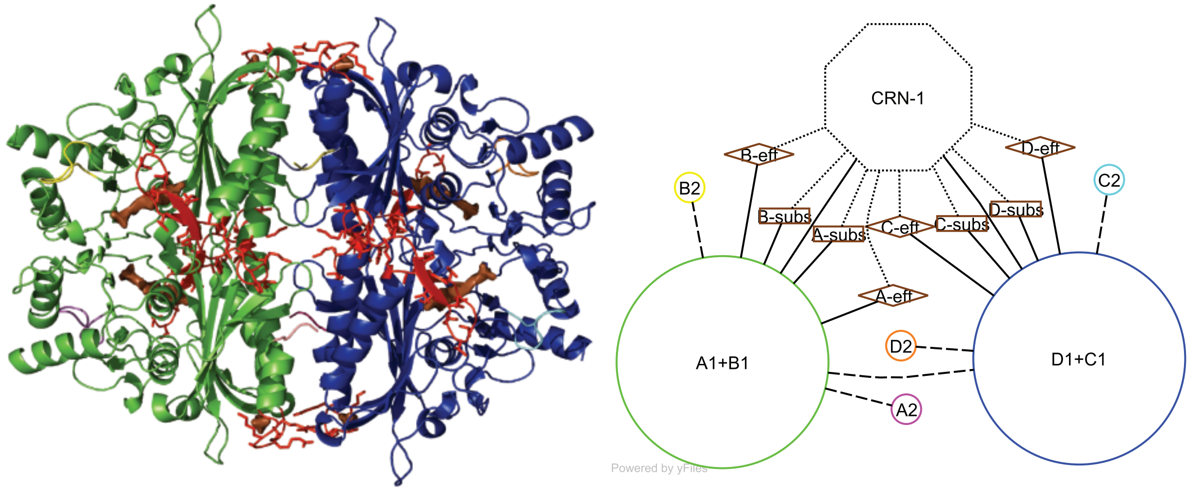

GTP cyclohydrolase I

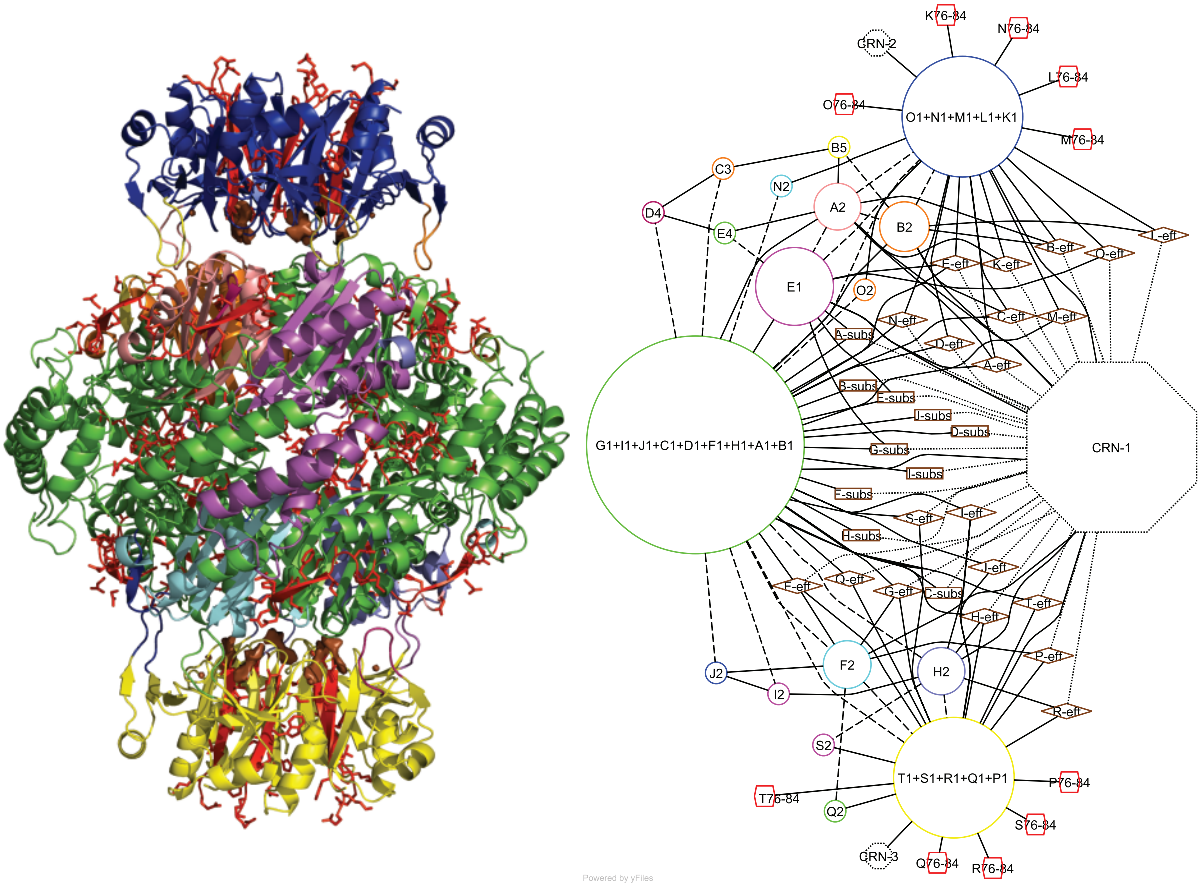

glcN-6-P deaminase

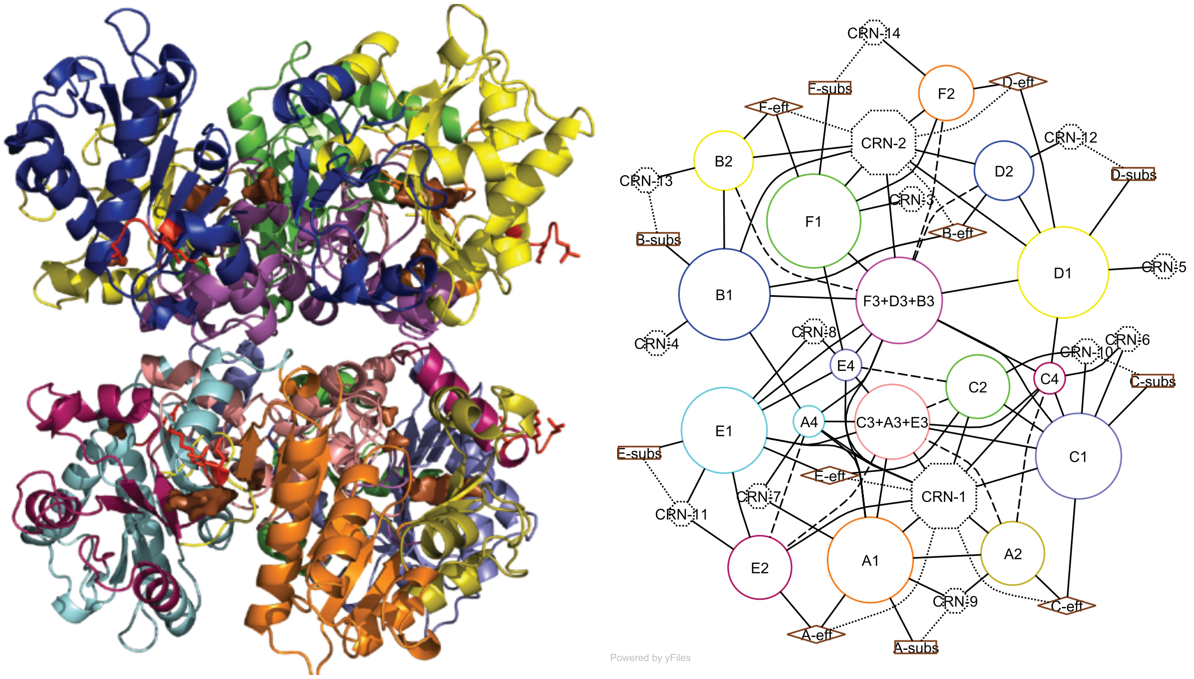

glycogen phosphorylase

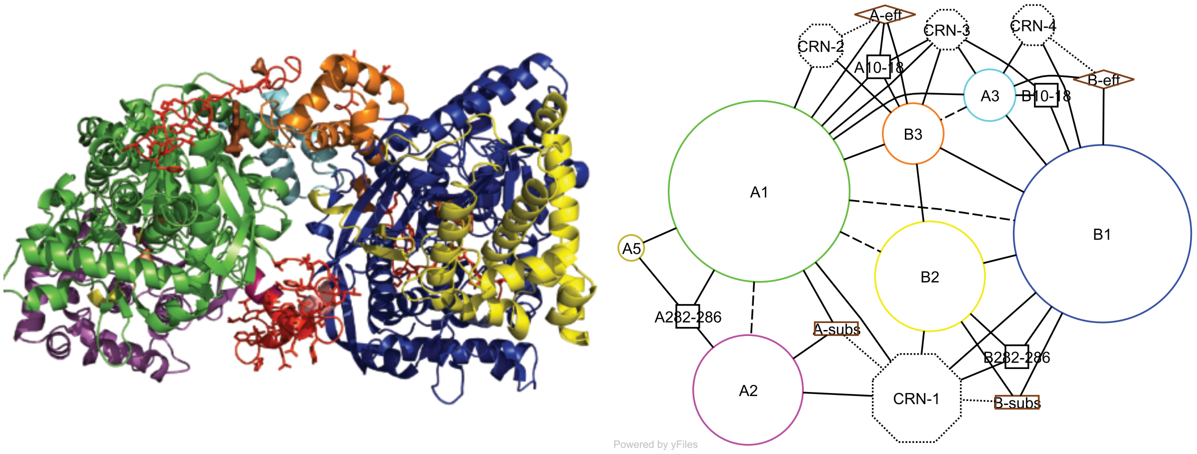

hemoglobin

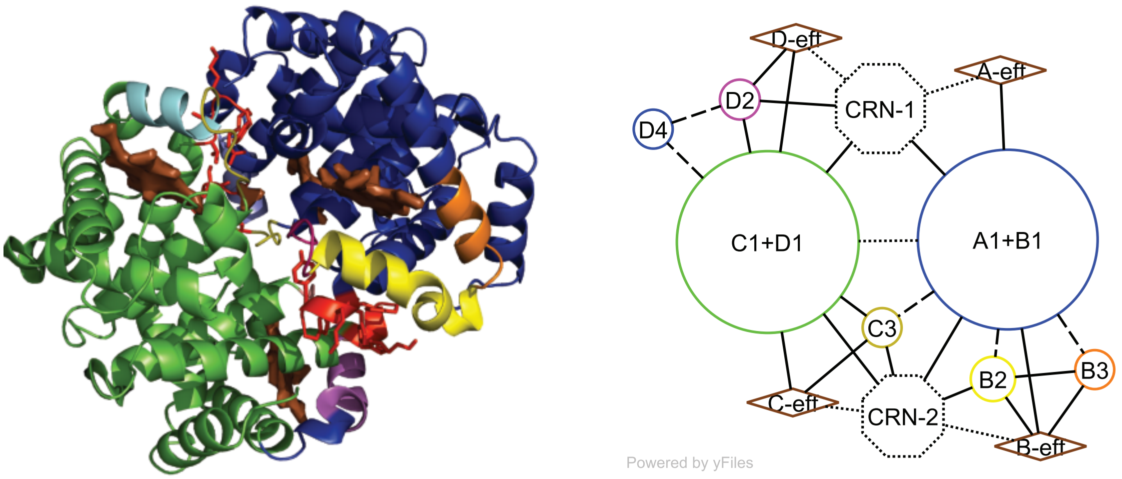

lactate DH

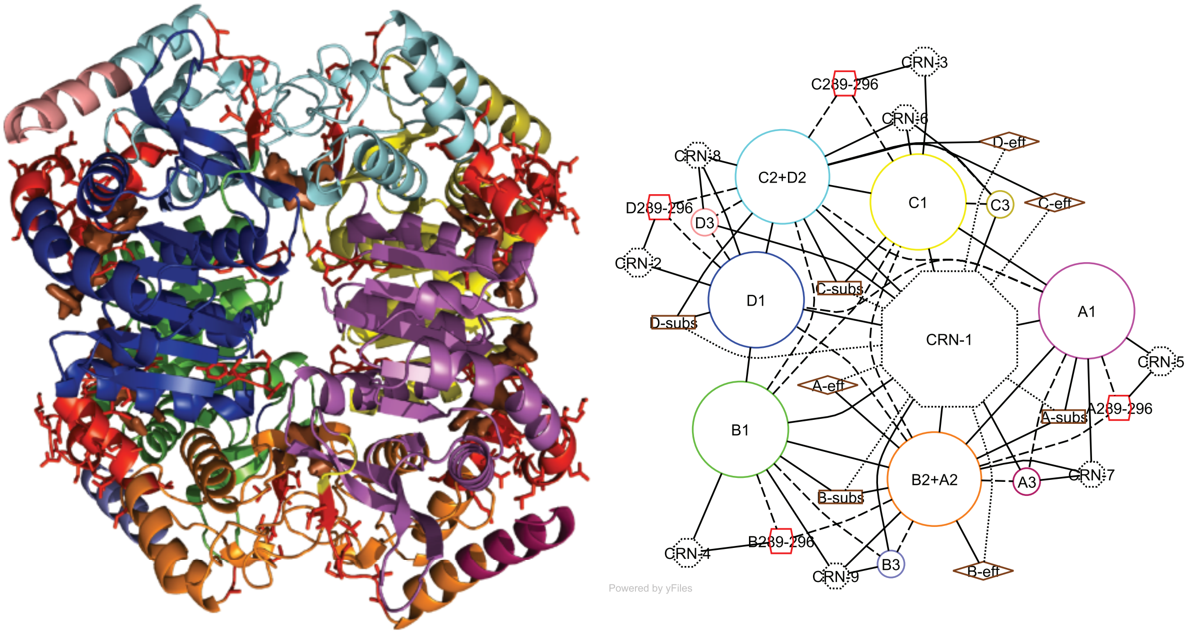

phosphoglycerate DH

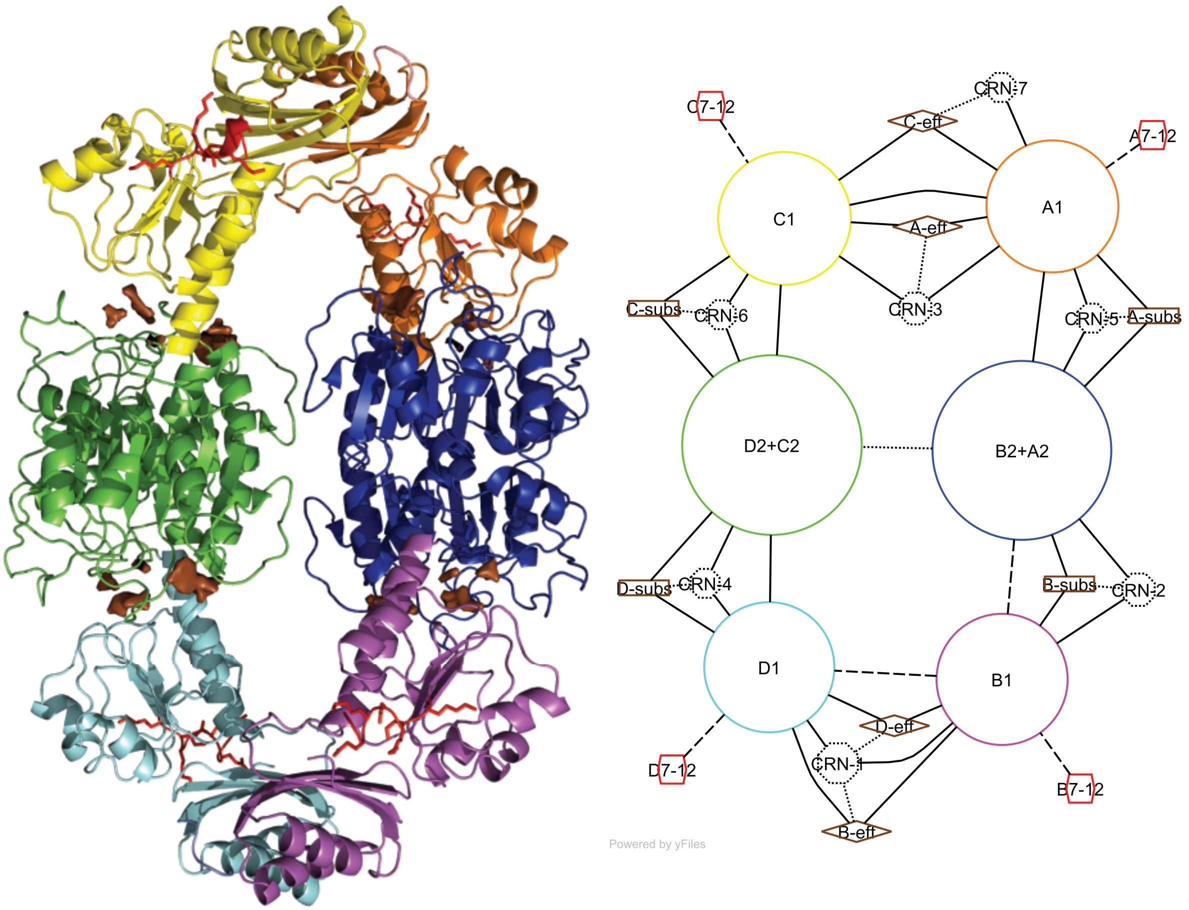

PTP1B

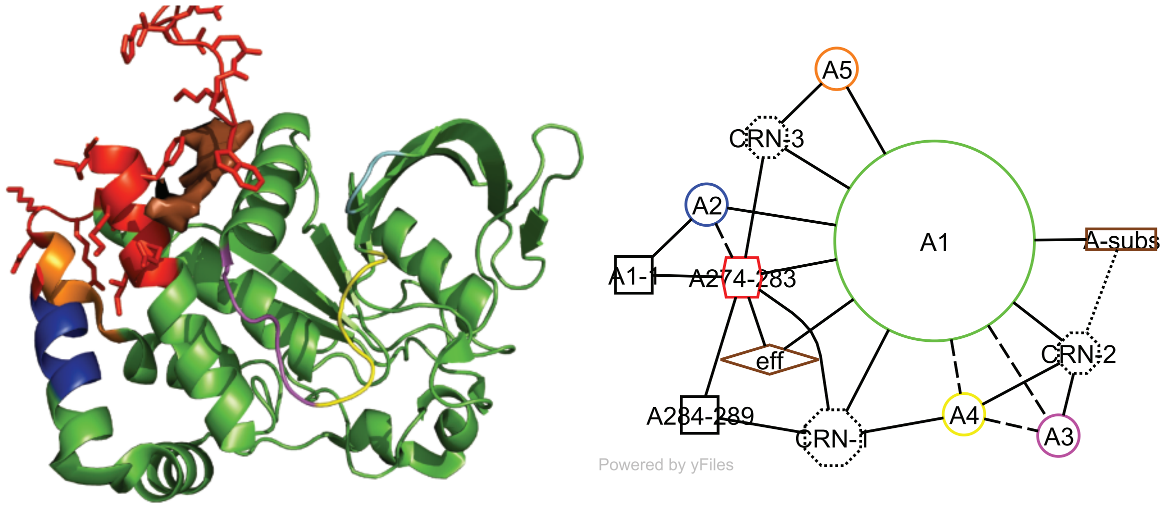

uracil PRT

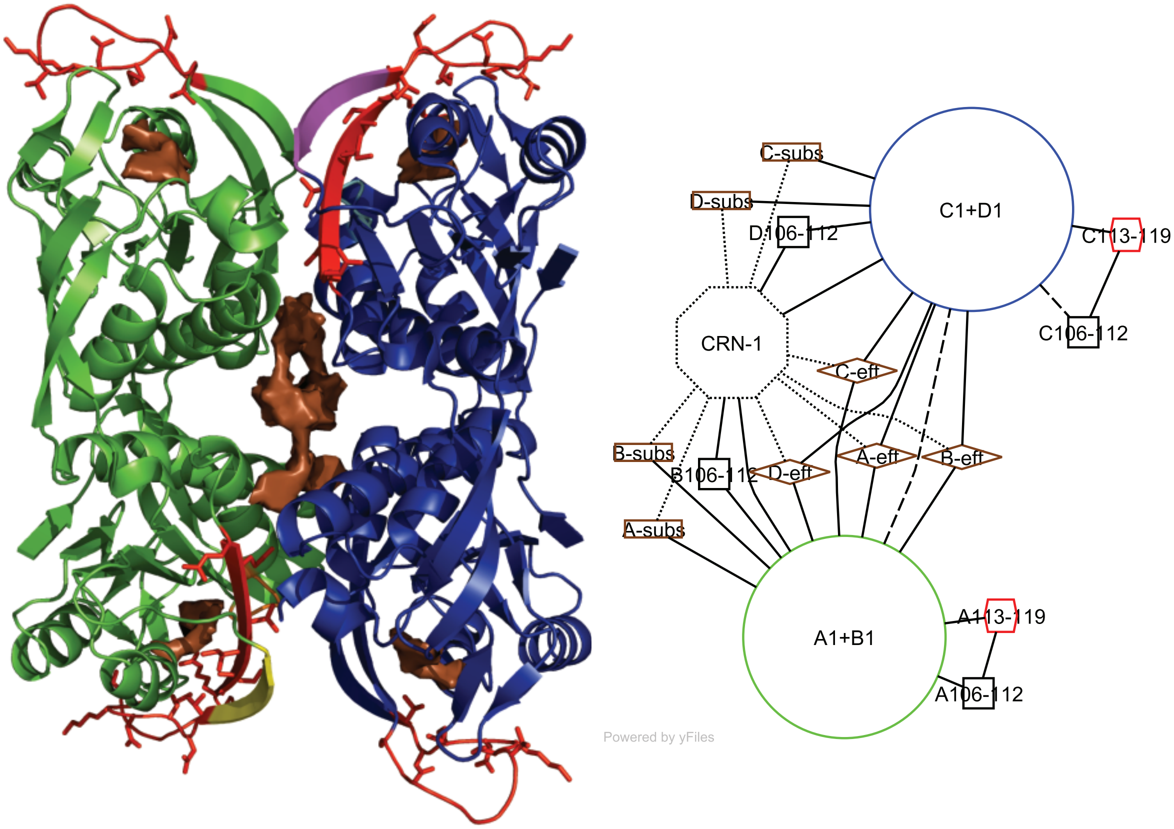

End Figure S2.
