# Supplementary material for: Allosteric Communication Occurs via Networks of Tertiary and Quaternary Motions in Proteins
Source: PLoS Comput Biol. 2009 Feb 20;5(2):e1000293. doi: 10.1371/journal.pcbi.1000293 (PMC2634971; doi:10.1371/journal.pcbi.1000293)
Supplement: Figure S3 — Proposed pathways in the GCNs of lac repressor and NAD-malic enzyme. The complete GCNs of these two proteins are shown in figure 4 of the main text, and pathways are calculated as in figure 5 of the main text. One path is shown per protein. A pathway connecting two effectors is shown for malic enzyme because there are no substrate-effector pathways in this protein. (0.04 MB PDF) [file pcbi.1000293.s004.pdf]

lac repressor (A-eff to DNA-E)

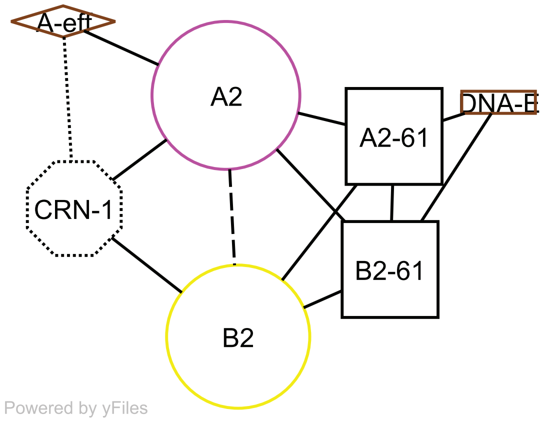

malic enzyme (A-eff to D-eff)

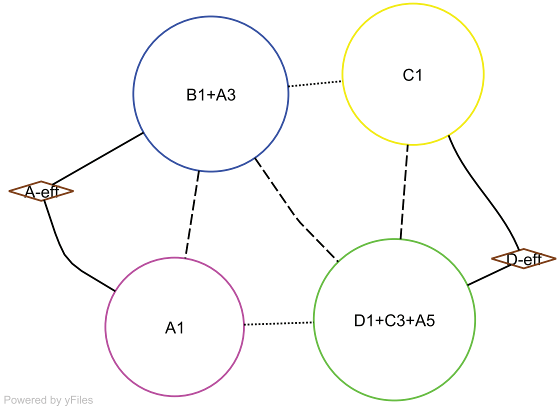

Figure S3: Proposed pathways in the GCNs of lac repressor and NAD-malic enzyme
The complete GCNs of these two proteins are shown in figure 4 of the main text, and pathways are calculated as in figure 5 of the main text. One path is shown per protein. A pathway connecting two effectors is shown for malic enzyme because there are no substrate-effector pathways in this protein.
