# Supplementary material for: Allosteric Communication Occurs via Networks of Tertiary and Quaternary Motions in Proteins
Source: PLoS Comput Biol. 2009 Feb 20;5(2):e1000293. doi: 10.1371/journal.pcbi.1000293 (PMC2634971; doi:10.1371/journal.pcbi.1000293)
Supplement: Protocol S1 — This file contains the supplementary methods referenced in the manuscript. (0.10 MB PDF) [file pcbi.1000293.s005.pdf]

Supplementary methods

Center of rotation

It might seem intuitive to calculate the translation from |**t**|, the displacement of c_S, the center of mass of S. However, except for translation parallel to **u**, **t** may arise from rotation about a physically meaningful axis that does not pass through c_S. We define an axis as physically meaningful if this axis passes near the interface between L and S, that is, the residues that define the 6 Å contacts between L and S. We define an axis as passing near a set of residues if the C_α atom of any residue in the set is no more than 5.0 Å from the axis. To assess this, we first find a center of rotation c_R, such that the motion can be described as a rotation by θ about **u**, centered at c_R, and a translation T_u parallel to **u**.

To do this, we first find the basis vectors $\mathbf{e_x}$, $\mathbf{e_y}$, and $\mathbf{e_z}$ of a right-handed orthogonal coordinate system centered at c_R describing the counterclockwise motion from state A to state B. Let s_1 and s_2 be the centers of S_A and S'_B, and let **s** be the vector between the two centers. Let $\mathbf{e_z} = \mathbf{u}$, and let $\mathbf{e_x}$ lie in a plane defined by $\mathbf{e_z}$ and the midpoint of **s** such that s_1 and s_2 are in the ($\mathbf{e_x}$,- $\mathbf{e_y}$) and ($\mathbf{e_x}$, $\mathbf{e_y}$) quadrants, respectively. Then, $\mathbf{e}_\mathrm{x} = \mathrm{unit}(\mathbf{s} \times \mathbf{e}_\mathrm{z})$, where $\mathrm{unit}(\mathbf{v}) = \mathbf{v}/|\mathbf{v}|$, and $\mathbf{e}_\mathrm{y} = \mathbf{e}_\mathrm{z} \times \mathbf{x}$.

Then, we find the distance of s_1 from the origin in the $\mathbf{e_y}$ and $\mathbf{e_x}$ directions. Since $\mathbf{e_x}$ lies in a plane bisecting **s**, s_1 is $y = \frac{1}{2}(\mathbf{s} \bullet \mathbf{e}_\mathrm{y})$ from the origin in the –$\mathbf{e_y}$ direction. Next, we triangulate to find x, the distance of s_1 from the origin in the $\mathbf{e_x}$ direction:

$$x = \frac{y}{\tan(\frac{1}{2}\theta)}.$$

To find c_R, we simply extrapolate back:

$$c_\mathrm{R} = s_1 - x\mathbf{e}_\mathrm{x} + y\mathbf{e}_\mathrm{y}.$$
