# Supplementary material for: Allosteric Communication Occurs via Networks of Tertiary and Quaternary Motions in Proteins
Source: PLoS Comput Biol. 2009 Feb 20;5(2):e1000293. doi: 10.1371/journal.pcbi.1000293 (PMC2634971; doi:10.1371/journal.pcbi.1000293)
Supplement: Table S1 — Homotropic and heterotropic connectivity in 22 proteins. “Tertiary network” refers to the contact rearrangement network and “quaternary network” refers to the quaternary subgraph of the global communication network (GCNQ). “Homo” refers to substrate-substrate and effector-effector paths, and “hetero” refers to substrate-effector paths. (0.05 MB PDF) [file pcbi.1000293.s006.pdf]

	% paths					
	tertiary		quaternary		global	
protein name	homo	het	homo	het	homo	het
AraC	0%	-	0%	-	0%	-
ArgR	100%	-	0%	-	100%	-
MetR	50%	0%	0%	0%	50%	0%
TetR	0%	0%	100%	100%	100%	100%
anthranilate synthase	29%	50%	29%	50%	100%	100%
ATP sulfurylase	0%	0%	33%	0%	100%	100%
ATP-PRT	30%	0%	50%	0%	100%	100%
ATCase	25%	0%	0%	0%	25%	0%
caspase	100%	-	0%	-	100%	-
chorismate mutase	0%	0%	100%	100%	100%	100%
DAHP synthase	50%	25%	50%	25%	100%	100%
FBPase-1	100%	100%	0%	0%	100%	100%
GTP cyclohydrolase I	100%	100%	40%	15%	100%	100%
glcN-6-P deaminase	20%	0%	50%	0%	100%	100%
glycogen phosphorylase	50%	0%	50%	50%	100%	100%
hemoglobin	33%	-	0%	-	100%	-
lactate DH	100%	100%	50%	0%	100%	100%
NAD-malic enzyme	17%	0%	50%	0%	50%	0%
phosphofructokinase	0%	25%	50%	25%	100%	100%
phosphoglycerate DH	8%	0%	100%	100%	100%	100%
PTP1B	-	0%	-	0%	-	100%
uracil PRT	100%	100%	50%	0%	100%	100%
hits (≥ 20% of paths)	70%	39%	70%	39%	96%	83%
hits (all paths)	26%	22%	22%	17%	83%	83%

Table S1: Homotropic and heterotropic connectivity in 22 proteins
"Tertiary network" refers to the contact rearrangement network and "quaternary network" refers to the quaternary subgraph of the global communication network (GCN_Q). "Homo" refers to substrate-substrate and effector-effector paths, and "hetero" refers to substrate-effector paths.
