# Supplementary material for: Allosteric Communication Occurs via Networks of Tertiary and Quaternary Motions in Proteins
Source: PLoS Comput Biol. 2009 Feb 20;5(2):e1000293. doi: 10.1371/journal.pcbi.1000293 (PMC2634971; doi:10.1371/journal.pcbi.1000293)
Supplement: Table S2 — Proteins with rms net cutoffs other than 0.8 Å. (0.01 MB PDF) [file pcbi.1000293.s007.pdf]

protein	rms_{net} cutoff
DAHP synthase	0.5
hemoglobin	0.5
phosphofructokinase	0.5
PurR	1.0
anthranilate synthase	1.0
ATP sulfurylase	1.0
ATP-PRT	1.0

Table S2: Proteins with rms_{net} cutoffs other than 0.8 Å
